# Assessment and diagnostic relevance of novel serum biomarkers for early decision of ST-elevation myocardial infarction

**DOI:** 10.18632/oncotarget.4001

**Published:** 2015-05-18

**Authors:** Hun-Jun Park, Ji Heon Noh, Jung Woo Eun, Yoon-Seok Koh, Suk Min Seo, Won Sang Park, Jung Young Lee, Kiyuk Chang, Ki Bae Seung, Pum-Joon Kim, Suk Woo Nam

**Affiliations:** ^1^ Department of Cardiology, College of Medicine, The Catholic University of Korea, Seoul, Republic of Korea; ^2^ Department of Pathology, College of Medicine, The Catholic University of Korea, Seoul, Republic of Korea; ^3^ Functional RNomics Research Center, College of Medicine, The Catholic University of Korea, Seoul, Republic of Korea; ^4^ Cancer Evolution Research Center, College of Medicine, The Catholic University of Korea, Seoul, Republic of Korea

**Keywords:** ST-segment-elevation myocardial infarction, peripheral blood transcriptome, molecular signature, diagnostic biomarkers

## Abstract

Blood transcriptome reflects the status of diseases, and characteristic molecular signature provides a novel window on gene expression preceding acute coronary events. We aim to determine blood transcriptome-based molecular signature of acute coronary syndrome (ACS), and to identify novel serum biomarkers for early stage ST-segment-elevation myocardial infarction (STEMI). We obtained peripheral blood from the patients with ACS who visited emergency department within 4 hours after the onset of chest pain: STEMI (*n* = 10), Non-ST-segment-elevation MI (NSTEMI, *n* = 10) and unstable angina (UA, *n* = 11). Blood transcriptome scans revealed that a characteristic gene expression change exists in STEMI, resulting in 531 outlier genes as STEMI molecular signature (Welch's *t* test, *P* < 0.05). Another analysis with a set of blood samples of patients with STEMI (*n* = 7) before and 7 days after the primary percutaneous coronary intervention (*n* = 7) and normal control (*n* = 10) evidenced that STEMI molecular signature directly reflects the onset of STEMI pathogenesis. From the two sets of transcriptome-based STEMI signatures, we identified 10 genes encoding transmembrane or secretory proteins that are highly expressed in STEMI. We validated blood protein expression levels of these 10 putative biomarkers in 40 STEMI and 32 healthy subjects by ELISA. Data suggested that PGLYRP1, IRAK3 and VNN3 are more specific and sensitive diagnostic biomarkers for STEMI than traditional CK-MB or troponin.

Blood transcriptome scans of ACS evidenced early stage molecular markers for STEMI. Here, we report novel biomarkers to diagnose STEMI at emergency department in hospitals by a simple ELISA method.

## INTRODUCTION

Advances in our understanding of the pathophysiology of acute coronary syndrome (ACS) have led to a marked increase in development of biomarkers for diagnosis, risk stratification, therapeutic decision-making, and assessment of clinical outcomes. Acute myocardial infarction (AMI), a type of ACS, is the most serious coronary artery disease (CAD), which can lead to cardiac death and/or heart failure [1]. According to the World Health Organization (WHO) guideline, AMI is defined by a combination of two of the three characteristics: 1) clinical history of ischemic type chest pain lasting for more than 20 minutes, 2) changes in serial electrocardiogram (ECG) tracings, and 3) rise and fall of serum cardiac biomarkers such as creatine kinase-MB (CK-MB) fraction and troponin [2]. The WHO criteria were refined in the year 2000 to give more prominence to cardiac biomarkers [3]. However, the use of cardiac biomarker for early diagnosis of AMI has limitations, which results in a lower quality care in the emergency department (ED), a higher rate of in-hospital mortality, and adverse long-term clinical outcomes [4, 5]. Therefore, recent efforts have been focused on finding more sensitive and specific surrogate markers to facilitate early installment of appropriate therapy in patients with AMI and to exclude ‘false-positive’ low-risk patients in the early hours after admission [6].

AMI is largely categorized into two categories: ST-segment-elevation myocardial infarction (STEMI) and non-ST-segment-elevation myocardial infarction (NSTEMI). STEMI is a condition in which the electrocardiographic ST is elevated above normal, while NSTEMI is characterized by the depression of ST segment or the inversion of the T-wave in the ECG. Another small category is unstable angina (UA), which has similar symptoms as NSTEMI [7].

The characteristic increase in serum biomarkers such as CK-MB and cardiac troponins (I, TnI and T, TnT) in AMI has caused researchers to consider these biomarkers as detectors of the cardiac condition [8]. However, while these biomarkers are extremely sensitive, the amount of time after the onset it takes for the levels of these biomarkers to rise to diagnostic sensitivity ranges from 12 to 140 hours, which does not fulfill the criterion as early surrogate markers.

Among the most easily accessible biofluids is the whole blood, containing leukocytes with informative transcripts used in their first line of immune defense and sentinels for many disease processes [9]. Using peripheral blood in clinical applications can also provide early and accurate information before development of the disease [10]. Therefore, blood gene expression profiling has the potential to be informative in disease status and of the underlying diverse disease mechanisms [11]. In recent studies, the use of blood-derived RNA has been facilitated to investigate genomic analyses. Thus, gene expression after AMI may reflect either triggering events or downstream consequences of plaque rupture and thrombosis, which suggests that transcriptional profiling may indicate mechanistic insights unbiased by preexisting disease hypotheses.

In the present study, we obtained peripheral blood from patients with ACS who visited emergency department within 4 hours after the onset of chest pain. We then profiled blood transcriptome from patients diagnosed with STEMI, NSTEMI, or UA via whole genome expression arrays. The blood transcriptomes were clustered to view characteristic molecular signature within ACS, confirming that STEMI has a unique expression signature even at very early time of disease event. STEMI molecular signature was then analyzed for secretory proteins to identify novel serum biomarkers that are informative and detectable for early diagnosis of STEMI from peripheral blood of patients.

## RESULTS

### Blood transcriptome scans of early stage ACS evidences that characteristic molecular signature exists in patients with STEMI

**Figure 1:**
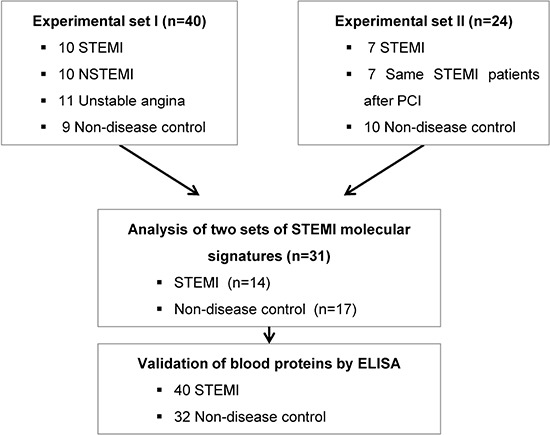
A schematic view of the procedure that contained patient cohorts and technologies used to identify novel biomarkers in peripheral blood of STEMI The blood transcriptome scans of ACS patient and normal healthy subjects were performed on two independent experimental sets. To identify the potent molecular signature of STEMI, combined analysis between two data sets was conducted. Then, blood protein levels of putative biomarkers in 40 STEMI and 32 normal samples were measured by ELISA.

**Figure 2:**
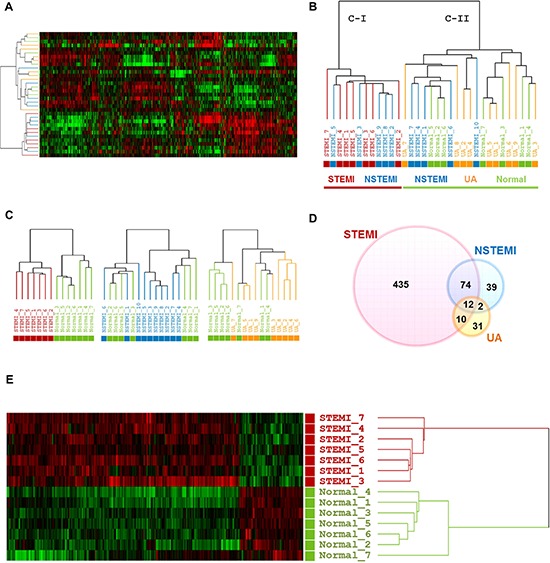
Transcriptome scans of onset of early stage ACS exhibited characteristic molecular signature in patients with STEMI **A.** and **B.**, Unsupervised hierarchical clustering of normal group and three categories of ACS. **C.**, Dendrograms derived from unsupervised clustering analysis of each ACS with normal group using the 8,945, 9,241 and 9,376 gene sets for STEMI, NSTEMI and UA, respectively. **D.**, Venn diagram showing overlap outlier genes between three different categories of ACS groups versus normal group. **E.**, Heatmap of the 531 DEGs between STEMI and normal group is shown. In this heat map, each strip represents a subject and each column represents the collection of genes. The intensity of each pixel is proportional to the relative gene expression levels in 1 sample. Upregulated genes are shown in red, downregulated genes are shown in green. Log ratios between the intensity of each sample and the baseline are shown.

Our primary question is whether molecular signature of peripheral blood from the early stage of ACS patients reflects different pathogenesis of ACS, providing novel window of gene expression changes as unique molecular signature for ACS. Thus, to tackle this issue, early ACS patients, namely those who visited emergency department within 4 hours after the onset of chest pain was used as the population for the transcriptome analysis. Figure 1 provides an overview of the study design. Firstly, a total of 40 blood samples (including STEMI, *n* = 10; NSTEMI, *n* = 10; UA, *n* = 9 and healthy subjects (*n* = 9) with normal coronary angiography) were subjected to whole genome expression microarray to profile ACS with its innate characteristic molecular signature. Of these, 7 samples (5 of ACS and 2 of normal) were excluded in the initial dataset due to low signal intensities or poor quality of RNA. In general, transcriptomic regulation is driven by dynamic intracellular responses to a biological stimulus. Thus, to investigate whether the large-scale gene expression changes in peripheral blood reflect different pathogenesis of ACS, we performed unsupervised hierarchical clustering analysis. As shown in Figure 2A, unsupervised hierarchical clustering analysis of the 33 samples with 9,737 genes that passed basic filtering criteria resulted in two main clusters within dendrogram: one cluster (C-I) that contains all of the STEMI (100%, 7/7) and a part of NSTEMI (50%, 5/10) patients, and a second cluster (C-II) that contains all of the normal (100%, 7/7), all of UA (100%, 9/9), and remaining NSTEMI patients (50%, 5/10) (Figure 2B). Whereas expression pattern of NSTEMI as well as UA were dispersed with the normal patterns, there was distinct boundary separating the STEMI samples (Figure 2C). This result indicated that large-scale gene expression changes associated with STEMI among ACS, and such gene expression changes may serve as unique molecular signature for the diagnosis or prediction of STEMI. In addition, we sought to identify differentially expressed gene set of each ACS group compared to normal group by using Welch's *t* test as described in Materials and Methods. The number of resulting differentially expressed genes in each ACS compared to normal group is shown in a Venn diagram (Figure 2D). The number of genes included in each gene set was 531, 127 and 55 genes for STEMI, NSTEMI and UA, respectively. Among the gene sets, 531 outlier genes for STEMI were able to clearly discern all STEMI from normal group without exception in hierarchical clustering analysis (Figure 1E).

### Validation of large-scale gene expression changes in blood transcriptome of patients with STEMI

**Figure 3:**
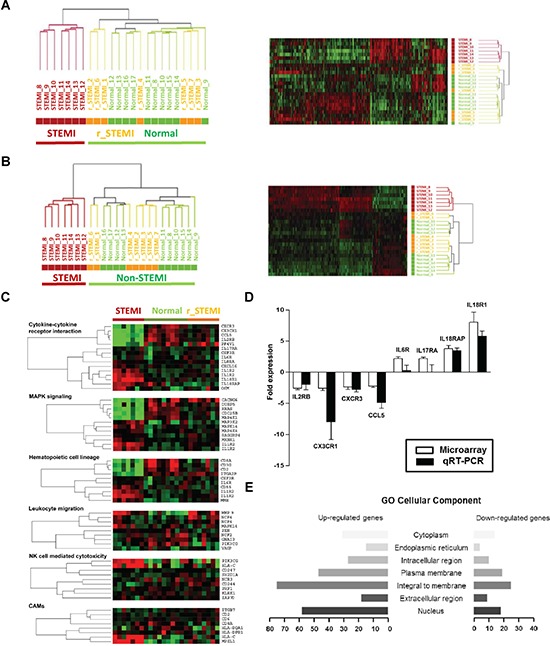
Large-scale gene expression changes are associated the pathogenesis of STEMI **A.**, Unsupervised hierarchical cluster analysis on the expression profiles of the 7 STEMI patients before (STEMI) and 7 days after PCI (r_STEMI), and 10 healthy controls (Normal). **B.**, Hierarchical cluster analysis of the 546 differentially expressed genes between STEMI and normal group. **C.**, Relevant biological themes annotating genes differentially expressed in the STEMI group compared to non-STEMI groups (r_STEMI and Normal) are indicated by categories from the KEGG definition. **D.**, Validation of the significance in gene expression changes from the microarray analysis by qRT-PCR analysis. **E.**, A bar graph of the cellular components of the STEMI molecular signature determined by the GO. Rows indicate the cellular components and the x-axis indicates the number of genes in the corresponding cellular component.

The fact that distinct STEMI-associated molecular signature exists in ACS transcriptomes led us to hypothesize that STEIMI-related gene expression changes might be responsible for STEMI pathogenesis. Thus, we investigated whether this signature disappeared from the blood transcriptomes of the patients who recovered from STEMI. To this end, we obtained peripheral blood from the patients with STEMI (*n* = 7) before and 7 days after the primary PCI and from healthy normal (*n* = 10). Unsupervised transcriptomic analysis of the three different groups (STEMI, after surgical operation group (r_STEMI) and normal) resulted in two main clusters; one cluster included all STEMI, whereas the other cluster including those recovered from STEMI and healthy subjects without exceptions (Figure 3A). This indicates that STEMI trasncriptome includes unique molecular signature which associated with STEMI pathogenesis providing novel molecular markers discerning STEMI within onset of acute coronary artery disease. To retrieve the maximum number of genes that precisely discriminate these two groups (STEMI vs non-STEMI group), we next performed Welch's *t* test to identify differentially expressed genes in STEMI patients compared to non-STEMI group. Supervised analysis of the Welch's *t* test (*P* < 0.05, 1.5-fold cut-off) resulted in 546 outlier genes that exactly separate the samples into two groups (Figure 3B and Supplemental Table 2).

To gain more insight into the possible mechanisms of STEMI-associated molecular signature, we sought to identify the pathways in which these 546 STEMI-signature genes participate. The probe sets of STEMI-associated molecular signature were classified into several groups of genes according to their biological functions using pathway finding algorithm of GSEA linked with KEGG pathway. Based on this database analysis, we identified 6 major pathways: cytokines/cytokine receptors, MAPK signaling, hematopoietic cell lineage, inflammatory leukocyte migration, natural killer cell function, and cell adhesion molecules (CAMs). Many of the gene elements classified as the cytokines-cytokine receptor interaction (10 of 15, 66.7%) were increased in expression level (Supplemental Table 3). For example, expressions of interleukin genes including interleukin 18 (*IL18R1* or *IL18RAP*), interleukin 1 (*IL1R2*), interleukin 17 (*IL17RA*), interleukin 6 (*IL6R*) and interleukin 8 (*IL8RA*) were significantly upregulated in STEMI compared with non-STEMI groups. Each of the six major functional groups of genes that showed differential expression is presented as heatmaps of one-way clustering analysis (Figure 3C).

Next, to validate gene expression data of microarrays and to confirm transcriptional levels of differentially expressed genes, we selected significantly deregulated genes from microarrays data of STEMI signature and performed qRT-PCR analysis. For a direct comparison, blood RNA from the same sets of patients used for microarray hybridization was used. Based on our microarray data, 8 differentially expressed genes were selected for this validation study: 4 up-regulated genes (*IL6R*, *IL17RA*, *IL18R1*, and *IL18RAP*) and 4 down-regulated genes (*CCL5*, *CX3CR1*, *CXCR3*, and *IL2RB*). Expression ratio (mean ± SD) of each gene was compared between microarray data and qRT-PCR results (Figure 3D). Lastly, the selected genes were also evaluated by hypergeometric distribution testing based on Gene Ontology (GO) annotations to determine their component within the cells (Figure 3E and Supplemental Table 4).

### Identification of gene elements encoding secretory proteins from the core molecular signature of STEMI transcriptome from peripheral blood

**Figure 4:**
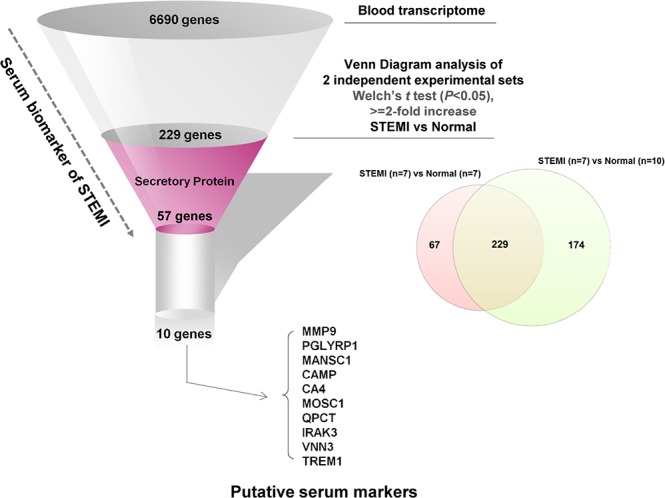
Flowchart for the strategy to dissect detectable biomarkers from peripheral blood of the patients with STEMI We extracted 229 differentially overexpressed outlier genes in STEMI group compared with those of normal group by joining of two sets of experiments, and identified the top 10 of the screened genes encoding transmembrane/secretory molecules and highly expressed in the blood transcriptome using SignalP 3.0 to conduct ELISA for clinical validation.

Blood transcriptomic analysis of patients with ACS indicated that STEMI-specific molecular signature reflected disease pathogenesis and provides large-scale gene elements that could be used as novel diagnostic biomarker for STEMI. Therefore, to identify potent molecular signature of STEMI, we retrieved gene elements that were commonly included as STEMI signature in the two different sets of independent experiments analyzed above (Experiment set I and Experiment set II of Figure 1). To perform further analysis, 6,690 gene elements were recapitulated as common probe set for these two experiments. Supervised analysis of the Welch's *t* test (>=2-fold, *P* < 0.05) algorithm resulted in 296 (Experiment set I) and 403 (Experiment set II) outlier genes, and then Venn Diagram analysis of these two outlier gene sets resulted in 229 gene elements that were commonly deregulated (Figure 4). Lastly, to identify detectable serum biomarkers from the peripheral blood of patients, 229 gene elements were subjected to the program SignalP 3.0 (http://www.cbs.dtu.dk/services/SignalP-3.0/) to select gene element harboring signal peptide indicating secretory protein. This series of analyses finally resulted in 57 gene elements that encode transmembrane or secretory molecules as novel biomarkers to diagnose STEMI at very early time of disease event. We selected 10 candidates that showed high expression in STEIM group compared to normal group; *MMP9* (9.3-fold), *PGLYRP1* (3.2-fold), *MANSC1* (4.2-fold), *CAMP* (3.9-fold), *CA4* (3.9-fold), *MOSC1* (3.5-fold), *QPCT* (3.4-fold), *IRAK3* (3.3-fold), *VNN3* (3.3-fold) and *TREM1* (3.2-fold) and quantified the plasma protein levels encoded by the candidate genes by way of ELISA test.

**Figure 5:**
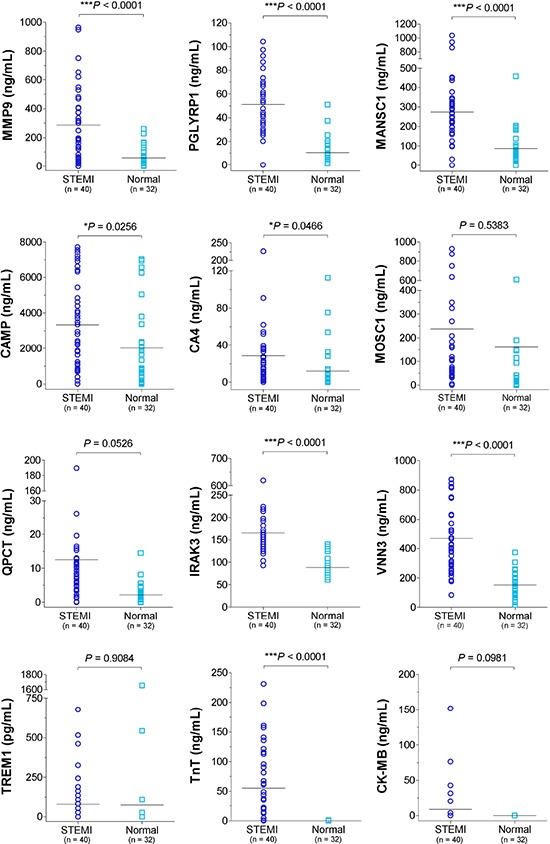
The plasma protein of the 10 candidate genes in patients with STEMI (*n* = 40) and normal group (*n* = 32) Expressions of 10 candidate gene product based on ELISA test presented as aligned dot plot, and the CK-MB, and TnT were used as positive control. (*, *P* < 0.05; ***, *P* < 0.001).

To this end, we obtained additional samples of STEMI patients (*n* = 40) and healthy normal controls (*n* = 32) and performed ELISA; mean door-to-balloon time was 80.4 ± 37.0 minute. Demographics of these validate cohort are shown in Table 1. The plasma levels of CK-MB and cardiac troponin T (TnT) which are well-known two markers of STEMI were detected and we found only TnT levels was significantly increased in this cohort of STEMI patients. Among putative serum biomarkers, we found significant increase in the plasma levels of MMP (*P* < 0.0001), PGLYRP1 (*P* < 0.0001), MANSC1 (*P* < 0.0001), CAMP (*P* = 0.0256), CA4 (*P* = 0.0466), IRAK3 (*P* < 0.0001), and VNN3 (*P* < 0.0001) in STEMI group compared to those of normal group, whereas the increases in MOSC1 (*P* = 0.5383), QPCT (*P* = 0.0526), and TREM1 (*P* = 0.9084) were not statistically significant (Figure 5).

**Table 1:** Baseline characteristics

Variables	STEMI	Control	*P* value
	(*n* = 40)	(*n* = 32)	
Demographics			
Age, years	63.7 ± 2.1	55.9 ± 2.5	0.02
Male, n (%)	22 (55.0)	19 (59.4)	0.71
Body mass index	23.5 ± 0.4	25.3 ± 0.8	0.04
Hypertension, n (%)	24 (60.0)	14 (43.8)	0.17
Diabetes, n (%)	12 (30.0)	4 (12.5)	0.08
Hyperlipidemia, n (%)	8 (20.0)	5 (15.6)	0.63
Current smoking	14 (35.0)	6 (18.8)	0.13
Laboratory findings			
WBC	10.0 ± 0.6	6.3 ± 0.3	< 0.01
Seg	63.2 ± 2.9	52.2 ± 2.6	0.01
Hemoglobin	13.5 ± 0.3	14.2 ± 0.3	0.09
Creatinine	1.1 ± 0.03	0.8 ± 0.1	0.03
TC	173.3 ± 6.5	171.5 ± 6.1	0.84
TG	104.4 ± 9.1	119.7 ± 10.3	0.27
HDL	44.2 ± 2.0	43.2 ± 1.7	0.73
LDL	106.8 ± 5.5	102.5 ± 5.7	0.6
hsCRP	3.0 ± 1.4	0.3 ± 0.2	0.07
CK-MB	8.7 ± 4.4	0.0 ± 0.0	0.05
TnT	55.0 ± 9.9	0.03 ± 0.03	< 0.01
Extent of CAD			
One vessel	12 (30.0)		
Two vessel	18 (45.0)		
Three vessel	10 (25.0)		
Culprit lesion, n (%)			
LAD	19 (47.5)		
LCX	4 (10.0)		
RCA	17 (42.5)		
Pre TIMI flow 0−1, n (%)	21 (52.5)		
Collateral 0−1, n (%)	38 (95.0)		

WBC, white blood cell; Seg, segmented neutrophil count; TC, total cholesterol; TG, triglyceride; HDL, high density lipoprotein cholesterol; LDL, low density lipoprotein cholesterol; CRP, complement reactive protein; CK-MB, creatin kinase-myocardial band; TnT, cardiac troponin T; CAD, coronary artery disease; LAD, left anterior descending artery; LCX, left circumflex artery; RCA, right coronary artery; TIMI, Thrombolysis in myocardial infarction.

**Figure 6:**
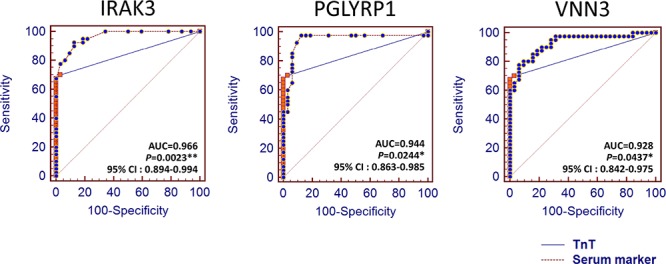
The ROC curve analysis of the plasma proteins encoded by the 3 candidate genes and STEMI standard marker, TnT Statistically significant difference of AUC compared with TnT (*, *P* < 0.05; **, *P* < 0.01).

Comparison analysis of receiver operating characteristic (ROC) curve was performed to determine more putative biomarker than the known biomarkers (Supplemental Figures 1, 2 and Supplemental Table 5). The areas under the curve (AUC) of IRAK3 (AUC, 0.966, *P* < 0.0023), PGLYRP1 (AUC, 0.944, *P* < 0.0244), and VNN3 (AUC, 0.928, *P* < 0.0437) were indicated to be significantly more sensitive and specific than that of TnT (AUC, 0.845) (Figure 6).

A logistic regression analysis model was constructed to rule out the effects of irrelevant factors including several univariate factors such as age, diabetes, WBC, seg, hemoglobin, and creatinine were significantly associated with STEMI as well as the levels of CAMP, IRAK3, MANSC1, MMP9, PGLYRP1, QPCT, and VNN3. After adjustment using a multivariate logistic regression analysis model, only WBC, IRAK3 and PGLYRP1 were determined to be significantly predictive for STEMI. The combined results from the ROC curve analysis and multivariate logistic regression indicated preoperative IRAK3 as the strongest independent predictor for STEMI (OR, 1.058, 95% CI, 1.021 to 1.10; *P* < 0.013) (Table 2).

**Table 2:** Logistic regression analysis identifying serum biomarkers for early decision of ST-elevation myocardial infarction

Variables	Univariate Factors		Multivariate Factors	
	OR (95% CI)	*P* value	OR (95% CI)	*P* value
Age, years	1.042 (1.005 − 1.081)	0.026	—	—
Gender, Male	1.196 (0.467 − 3.065)	0.710	—	—
Body mass index	0.811 (0.656 − 1.002)	0.052	—	—
Hypertension	0.519 (0.202 − 1.330)	0.172	—	—
Diabetes	0.333 (0.096 − 1.160)	0.084	—	—
Hyperlipidemia	0.741 (0.217 − 2.532)		—	—
Current smoking	0.429 (0.143 − 1.288)	0.131	—	—
WBC	2.125 (1.429 − 3.160)	< 0.0001	1.913 (0.925 − 3.955)	0.08
Seg	1.040 (1.008 − 1.073)	0.013	—	—
Hemoglobin	0.793 (0.603 − 1.044)	0.098	—	—
Creatinine	19.919 (2.040 − 194.488)	0.010	—	—
TC	1.001 (0.988 − 1.015)	0.839	—	—
TG	0.995 (0.985 − 1.004)	0.273	—	—
HDL	1.008 (0.963 − 1.056)	0.724	—	—
LDL	1.004 (0.988 − 1.021)	0.596	—	—
hsCRP		0.444	—	—
CK−MB	—	—	—	—
TNT	3.155 (0.303 − 32.867)	0.337	—	—
CAMP	1.000 (1.000 − 1.000)	0.030	—	—
CA4	1.022 (0.999 − 1.046)	0.066	—	—
IRAK3	1.090 (1.051 − 1.130)	< 0.0001	1.058 (1.012 − 1.10)	0.013
MANSC1	1.011 (1.005 − 1.017)	< 0.0001	—	—
MMP9	1.596 (1.229 − 2.072)	< 0.0001	—	—
MOSC1	1.000 (0.999 − 1.001)	0.554	—	—
PGLYRP1	1.156 (1.082 − 1.236)	< 0.0001	1.075 (0.997 − 1.160)	0.06
QPCT	1.321 (1.136 − 1.536)	< 0.0001	—	—
TREM	1.000 (0.998 − 1.002)	0.907	—	—
VNN3	1.018 (1.009 − 1.027)	< 0.0001	—	—

OR (95% CI) indicates odds ratio (95% confidence interval).

## DISCUSSION

Percutaneous coronary intervention is one of the most prominent treatments for patients with STEMI. The door-to-balloon time should be less than 90 minutes, because any delay in time of reperfusion after arrival at the hospital is associated with a higher risk of in-hospital mortality [12]. Such mortality would decrease dramatically with the introduction of more sensitive and specific biomarkers for early identification of STEMI. However, there are substantial obstacles for achieving this goal that involve issues with transportation, hospital facilities, admission wait times, and diagnostic testing. Because time delays contribute to poor outcomes, improvements that lead to reduced time to diagnosis and therapy continue to be sought, especially with respect to STEMI, where biomarkers serve as the main diagnostic decision point. In general, it is believed that prior to the onset of dysfunctional effects in ACS, effects are already present at the cellular level. Impact at the cellular level depends on the type, magnitude, and duration of ischemic cardiovascular diseases, and may be reflected by the activations of different pathogenesis of ACS, which suggests that analysis of molecular stress responses in exposed blood cells can produce information regarding each mode of action and mechanisms underlying the different pathogenesis of ACS. Since molecular stress responses are reflected by changes in gene expression, we challenged that the peripheral blood transcriptome of different category of ACS is able to discriminate different pathogenesis of ACS and provide a novel window of gene expression changes as unique molecular signature for ACS.

From the onset of STEMI, genome expression, molecular, cellular and interstitial changes take place and biomarkers representing these biological pathways are now becoming recognized as potent predictors for the acute phase of STEMI. In several recent studies, the whole blood RNA was used as a source of extracting blood gene expression patterns based on microarrays or RT-PCR [13, 14]. However, no study has yet to identify novel biomarkers from the peripheral blood patients with ACS. Many of previous reports suggested that several intracellular inflammatory responses have crucial roles in ischemic cardiovascular diseases, which are yielded by neutrophil infiltration and leukocyte rolling followed by the cytotoxic damage to cardiomyocytes [15]. Therefore, we hypothesized that peripheral blood transcripome scans could provide a very sensitive and specific window for verifying ischemic heart diseases such as STEMI. Our results prove that blood transcriptome of the patients with STEMI has unique expression pattern among ACS, while NSTEMI is molecularly heterogeneous and is located on the border between the normal and acute myocardial infarction stage. Furthermore, additional experiment showing different blood transcriptomes between patients with STEMI and disease-free after PCI treatment indicated that STEMI-associated large-scale gene expression changes are caused by specific pathogenesis of STEMI (Figure 3).

With regards to specific putative biomarkers, we found 10 candidates that encode transmembrane or secretory molecules. Among these, we observed significant increase in the plasma levels of MMP, PGLYRP1, MANSC1, CAMP, CA4, IRAK3, and VNN3 in patients with STEMI groups by ELISA test (Figure 5). While cardiac troponins serve as a current key criterion for the diagnosis of AMI, diagnostic information is also obtained from serum CK-MB, total CK, and myoglobin (MYO) concentrations. However, our results demonstrated that only TnT levels was significantly increased in early phase of peripheral blood with STEMI patients. Notably, we also found that IRAK3, PGLYRP1, and VNN3 were significantly more sensitive and specific than that of TnT in ROC analysis providing novel cardiac biomarkers (Figure 6). The IL-1 receptor-associated kinase 3 gene (*IRAK3*) encodes a protein that negatively regulates Toll-like receptor signaling pathways involved in innate host defense and in the control of adaptive immune responses [16]. Short-type peptidoglycan-recognition protein 1 gene (*PGLYRP1*) encode an innate immunity protein that directly breaks down the structure of microbial cell wall PGNs, plays an important role both in antibacterial defenses and several inflammatory diseases [17]. The Vanin family represents a novel class of secreted or membrane-associated ectoenzymes, and *VNN3* transcripts are likely to be produced specifically in leukocytes (possibly neutrophil) and its expression might be regulated by a tissue-specific manner as well as TnT and erythrocyte protein [18]. Ironically, they are the most sensitive STEMI biomarkers, none has been reported for cardiovascular disease or cardiac markers.

In conclusion, transcriptome scans of peripheral blood from the patients with ACS revealed that STEMI was uniquely endowed with characteristic large-scale gene expression changes harboring pathogenic molecular signature, then gene elements encoding secretory molecules were dissected and validated for novel serum biomarkers which are informative and detectable for early diagnosis of STEMI from peripheral blood of patients. Here, we suggest a new set of novel biomarkers including IRAK3, PGLYRP and VNN3, which allow the diagnosis of STEMI at emergency department by a simple ELISA method.

## MATERIALS AND METHODS

### Patients and sample collection

Patients with ACS who underwent primary percutaneous coronary intervention (PCI) at the Seoul St. Mary's Hospital in South Korea within 4 hours after the onset of chest pain were enrolled in an institutional review board-approved protocol. Written informed consent was obtained from all subjects, and the study was approved by Ethics Committee of the Catholic University of Korea, College of Medicine (IRB approval number; CUMC10U037). As a control group, healthy subjects with normal coronary angiography were included in this study and all participants provided written consent. Blood samples were obtained from the arterial sheath before angiography and collected in PAXgene Blood RNA tube (Qiagen, Valencia, CA) for RNA isolation and in Vacutainer^®^ SST™ Serum Separation Tubes (BD, Franklin Lakes, NJ) for the enzyme-linked immunosorbent assay (ELISA), respectively.

### RNA isolation and blood whole genome expression analysis

Total RNA from blood was extracted according to optimized methods described previously with slight modifications [14, 19]. Briefly, whole blood (2.5 mL per patient) was collected directly into PAXgene Blood RNA tube, labeled with a unique identification number, stored at room temperature, and transferred to the laboratory within 4 hours for blood processing. Total RNA from blood was extracted using the PAXgene blood RNA kit (Qiagen) and purified with RNeasy kit (Qiagen) according to the manufacturer's instruction. The quality of total RNA was analyzed with the RNA StdSens Chips on the Experion™ system (BioRad, Hercules, CA). Microarray analysis was performed using Sentrix HumanRef-6 Expression BeadChip or HumanHT-12 v3 Expression BeadChip (Illumina, Inc., San Diego, CA). Approximately 37,000 genes with unique probe IDs were common to both platforms and were used for combined data analysis. The RNA was processed with Illumina RNA Amplification Kit (Ambion, Inc., Austin, TX) according to the manufacturer's instructions starting with 800 ng total RNA. Resulting biotin-labeled cRNA was recovered and purified with RNeasy kit (Qiagen), hybridized to the beadchips, and fluorescently tagged and scanned with Illumina BeadStation (Illumina) according to the manufacturer's protocol. All arrays were run in the same core facility. Gene expression microarray data set was submitted to the National Center for Biotechnology Information (NCBI), the Gene Expression Omnibus (GEO) database (http://www.ncbi.nih.gov/geo/), and the accession number is GSE61145.

### Blood transcriptome data analysis

Data analysis for STEMI blood transcriptome was performed using the following softwares: GenomeStudio (version 3.0, Illumina), GenPlex™ (version 3.0, ISTECH, Inc., Seoul, Korea), EXCEL (Microsoft), GSEA (version 2.07, Broad Institute), and SignalP (version 3.0., CBS). Briefly, GenomeStudio (version 3.0) was used for the data acquisition and calculation of signal values on Illumina expression beadchip. Normalization of expression data and hierarchical clustering were performed by GenPlex™ (version 3.0). For primary data filtering, spots with a P-call (Detection call *P*-value < 0.1) were selected, and normalized via quantile normalization.

A multitude of analyses was performed using the normalized and filtered data. Sets of differentially expressed genes (DEGs) were identified by combination analysis of Welch's *t* test and fold change, and the DEGs with a fold change deregulation of more than 2 and *P*-value < 0.05 were selected. To identify molecular pathways associated with significant DEGs in STEMI patients, pathway analysis was performed using the Kyoto Encyclopedia of Genes and Genomes (KEGG) pathway database (http://www.genome.jp/kegg/). Also, to investigate STEMI-specific genes that are enriched into the known molecular databases, Gene Set Enrichment Analysis (GSEA) was conducted using the standard procedures (http://www.broadinstitute.org/gsea). Briefly, the set of microarray data was analyzed for enrichment in two gene sets (GO Cellular Component and KEGG) related to the cellular component and biological pathway. As the output, GSEA provides a nominal *P* value for each gene set, which represents how significantly up- or down-regulated the genes within that set are in the microarray data comparison. To focus our attention on novel secretory proteins that the microarray data predict are upregulated in STEMI patients, the program SignalP 3.0 (http://www.cbs.dtu.dk/services/SignalP-3.0/) was used to identify signal peptides indicating secretion [20].

### Verification of gene expression with quantitative real-time reverse transcription-PCR

Reverse transcription was performed with total RNA isolated from whole blood and processed with Transcriptor first strand cDNA synthesis kit (Roche Applied Science, Indianapolis, IN). Quantitative real-time reverse transcription-PCR (qRT-PCR) was performed via SYBR green method using IQ5 RT-PCR detection system (Bio-Rad, Hercules, CA). The reaction mix (25 μL final volume) consisted of 12.5 μL of iQ SYBR Green Supermix (Bio-Rad), 2.5 μL of each primer (250 nM final concentration), 2.5 μL of H2O, and 5 μL of a 1/20 dilution of the cDNA preparation. Primers were designed using Primer-BLAST (http://www.ncbi.nlm.nih.gov/tools/primer-blast/index.cgi?LINK_LOC=BlastHome) and the sequences are listed in Supplemental Table 1. The thermocycling program consisted of one hold at 95°C for 4 min, followed by 40 cycles of 10 s at 95°C and 45 s at 56°C. After completion of these cycles, melting-curve data was then collected to verify PCR specificity, contamination and the absence of primer dimers. Relative gene expressions were calculated via 2^−ΔΔCt^ method, in which Ct indicates cycle threshold, the fractional cycle number where the fluorescent signal reaches detection threshold [21]. Each sample was tested in duplicate in a 96-well plate.

### Enzyme-linked immunosorbent assay (ELISA)

Stored serum obtained from each categorical subject was thawed and assayed for 10 proteins predicted as STEMI-specific biomarkers (MMP9, PGLYRP1, MANSC1, CAMP, CA4, MOSC1, QPCT, IRAK3, VNN3, TREM1) using of a commercially available ELISA kit (Uscnk Life Science Inc., China). Assays for the well-known standard cardiac markers (CK-MB, TnT) were used as positive control.

### Statistical analysis

All statistical analyses were performed with the MedCalc version 12.1.4.0 (MedCalc Software, Mariakerke, Belgium). Receiver operating characteristic curve (ROC) analysis was constructed to assess the sensitivity and specificity of serum biomarkers and to determine the ability of the various parameters to discriminate STEMI patients. The method of DeLong et al was used for the calculation of standard error of the areas under the ROC curves (area under the curve, AUC) and of the difference between two AUCs [22]. *P*-values less than 0.05 were considered statistically significant. Continuous variables are reported as mean ± SE and interquartile range. Categorical variables are reported as number and percentages. Univariate and multivariate logistic regression analyses were performed to identify preoperative independent predictors for STEMI patients. All preoperative predictor variables that were identified as significant at a two-tailed nominal probability value of less than 0.05 in univariate regression analyses were then entered into a multivariate logistic regression analysis model.

## SUPPLEMENTARY FIGURES AND TABLES






