# Supplementary material for: Assessment and diagnostic relevance of novel serum biomarkers for early decision of ST-elevation myocardial infarction
Source: Oncotarget. 2015 May 18;6(15):12970–83. doi: 10.18632/oncotarget.4001 (PMC4536992; doi:10.18632/oncotarget.4001)
Supplement: Supplementary file 1 [file oncotarget-06-12970-s001.pdf]

SUPPLEMENTARY FIGURES AND TABLES

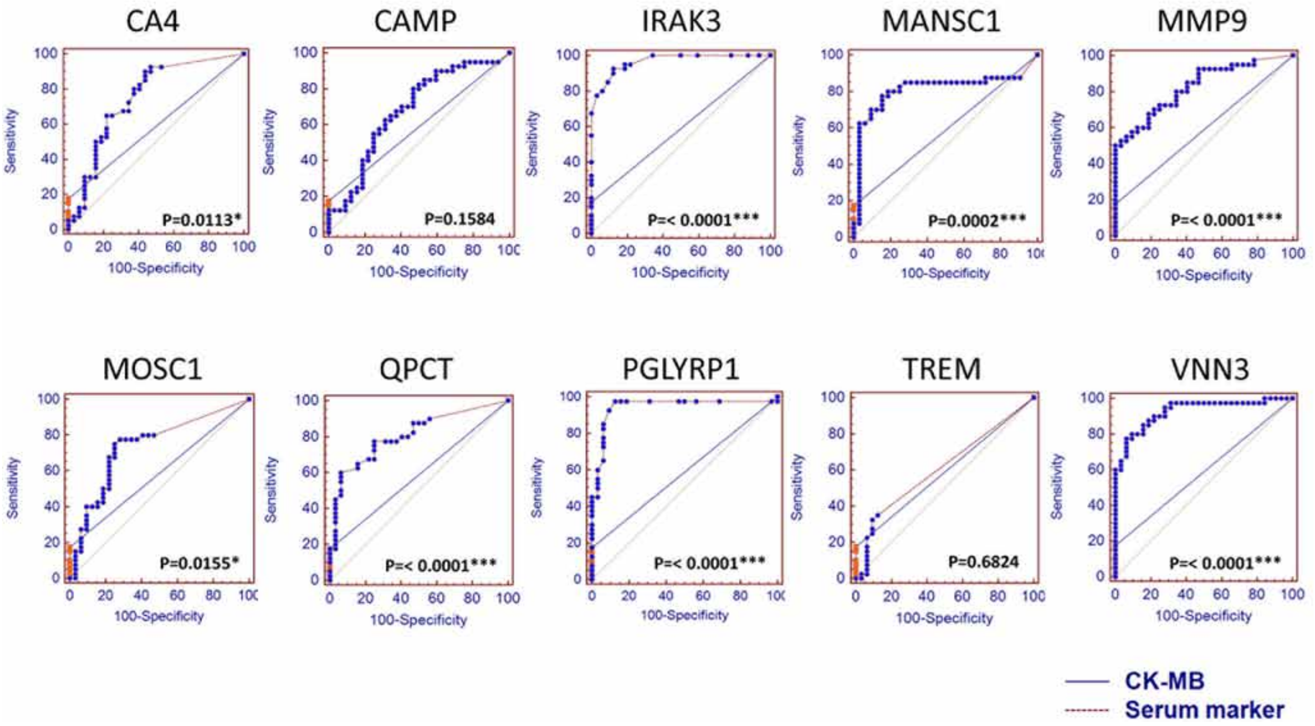

Supplementary Figure S1: The ROC curve analysis of the plasma proteins encoded by the 10 candidate genes and STEMI standard marker, CK-MB. Statistically significant difference of AUC compared with CK-MB (*, P < 0.05; **, P < 0.01; ***, P < 0.001).

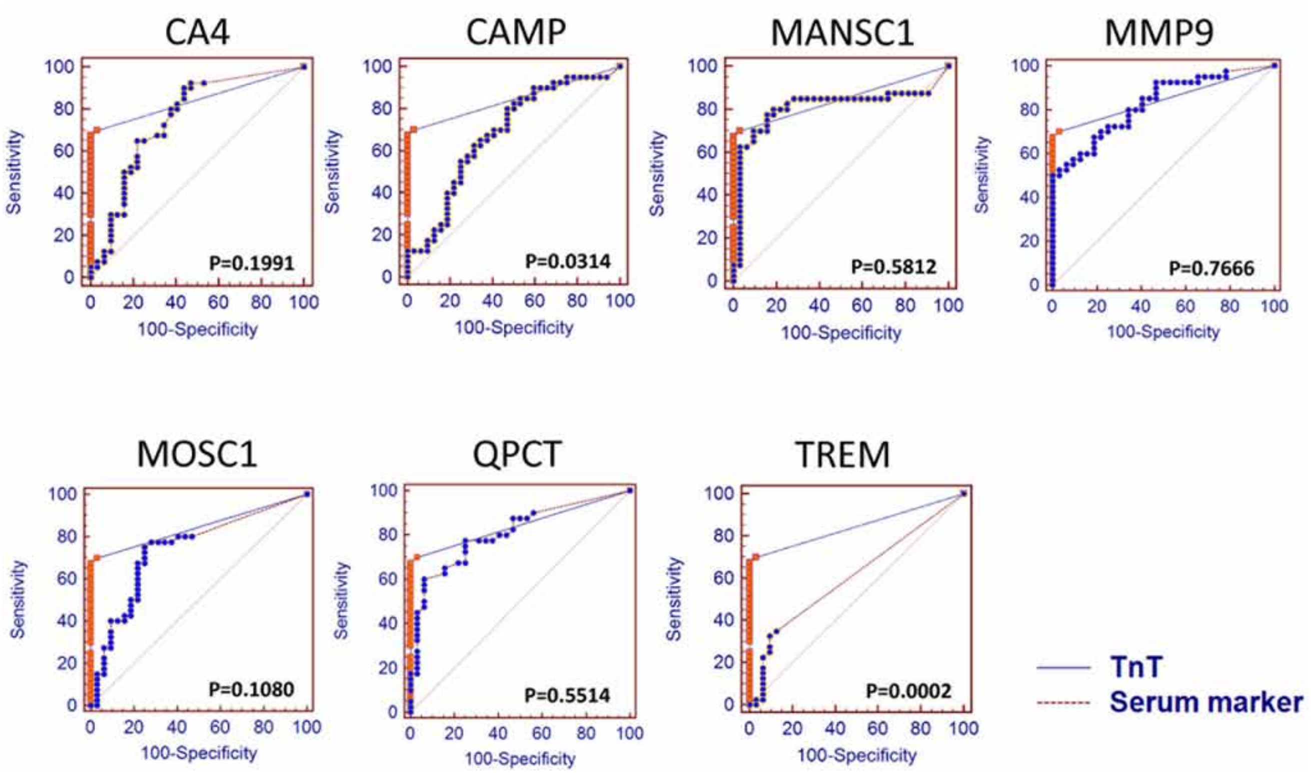

Supplementary Figure S2: The ROC curve analysis of the plasma proteins encoded by other 7 candidate genes and STEMI standard marker, TnT. Statistically significant difference of AUC compared with TnT. Although TREM and CAMP have statistically significant difference of AUC compared with TnT, we do not indicate then as markers since their AUC values are less than TNT.

Supplementary Table S1. Primer sequences used for quantitative real-time polymerase chain reaction

Gene symbol	GenBank Accession	Gene name	Forward primer 5′ –> 3′	Reverse primer 5′ –> 3′	Amplicon size (bp)
IL2RB	NM_000878.2	interleukin 2 receptor, beta	GAGACGTCC AGAAGTGGCTC	GTAACCCTGGT TGGTGAAGC	200
CX3CR1	NM_001337.3	chemokine (C-X3-C motif) receptor 1	TGACTGGCAG ATCCAGAGGTT	GTAGAATATGG ACAGGAACAC	164
CCL5	NM_002985.2	chemokine (C-C motif) ligand 5	CGCTGTCATC CTCATTGCTA	GACAAAGACG ACTGCTGGGT	172
CXCR3	NM_001504.1	chemokine (C-X-C motif) receptor 3, transcript variant A	ACCCAGCAG CCAGAGCACC	TCATAGGAAGA GCTGAAGTTCTCCA	111
IL6R	NM_000565.2	interleukin 6 receptor, transcript variant 1	TGCCATTGTT CTGAGGTTCA	TCGAGGTATT GTCAGACCCC	185
IL17RA	NM_014339.4	interleukin 17 receptor A	AATGAACGTT TGTGCGTCAG	CTCACAGTCA GGCACAAGGA	195
IL18RAP	NM_003853.2	interleukin 18 receptor accessory protein	GATCAGACGC TTGGGGATAA	CCTGGAGCC ACATCTCTTTC	185
IL18R1	NM_003855.2	interleukin 18 receptor 1	TGGAGGAGCT GTTGTTGATG	ATTGGGGCA AGAATGTGAAG	200
**Reference genes**					
GAPDH	NM_001256799.2	glyceraldehyde-3-phosphate dehydrogenase	AATGGGCAGCC GTTAGGAAA	GCGCCCAAT ACGACCAAATC	168

Supplementary Table S2. 546 Differentially expressed genes in STEMI compared with non-STEMI group (Normal and r_STEMI)

Supplementary Table S3.Differentially regulated gene transcripts (>2-fold) in STEMI compared with normal patients

GenBank ID	Gene symbol	Descriptive name	Fold change
Cytokine-cytokine receptor interaction (15)			
NM_002620.2	PF4V1	Platelet factor 4	− 2.75
NM_000878.2	IL2RB	Interleukin 2 receptor, beta	− 2.53
NM_001337.3	CX3CR1	Chemokine (C-X3-C motif) receptor 1	− 2.43
NM_002985.2	CCL5	Chemokine (C-C motif) ligand, other	− 2.17
NM_001504.1	CXCR3	Chemokine (C-X-C motif) receptor 3	− 2.14
NM_000634.2	IL8RA	Interleukin 8 receptor, alpha	2.00
NM_000565.2	IL6R	Interleukin 6 receptor	2.05
NM_156038.2	CSF3R	Colony-stimulating factor 3 receptor (granulocyte)	2.09
NM_022059.1	CXCL16	Chemokine (C-X-C motif) ligand 16	2.13
NM_014339.4	IL17RA	Interleukin 17 receptor	2.15
NM_020530.3	OSM	Oncostatin M	2.80
NM_004633.3	IL1R2	Interleukin 1 receptor, type II	3.39
NM_003853.2	IL18RAP	Interleukin 18 receptor accessory protein	3.58
NM_173343.1	IL1R2	Interleukin 1 receptor, type II	4.34
NM_003855.2	IL18R1	Interleukin 18 receptor 1	7.20
MAPK signaling pathway (12)			
NM_031897.2	CACNG6	Calcium channel, voltage-dependent, Gamma subunit 6	− 3.06
NM_021872.2	CDC25B	Cell division cycle 25B	− 2.06
NM_006270.3	RRAS	Ras-related protein R-Ras	− 2.06
NM_001042600.1	MAP4K1	Mitogen-activated protein kinase kinase Kinase kinase 1	− 2.04
NM_004419.3	DUSP5	Dual specificity phosphatase	− 2.04

(*Continued*)

GenBank ID	Gene symbol	Descriptive name	Fold change
NM_170604.2	RASGRP4	Ras guanyl nucleotide-releasing protein	2.03
NM_003684.3	MKNK1	MAP kinase interacting serine/threonine kinase	2.14
NM_145686.2	MAP4K4	Mitogen-activated protein kinase kinase kinase kinase 4	2.17
NM_001315.1	MAPK14	p38 MAP kinase	2.87
XM_001128799.1	MAP3K2	Mitogen-activated protein kinase kinase kinase 2	3.33
NM_004633.3	IL1R2	Interleukin 1 receptor, type II	3.39
NM_173343.1	IL1R2	Interleukin 1 receptor, type II	4.34
Hematopoietic cell lineage (10)			
NM_000073.1	CD3G	CD3G antigen, gamma polypeptide	− 2.27
NM_171827.2	CD8A	CD8A antigen, alpha polypeptide	− 2.26
NM_000419.3	ITGA2B	Integrin alpha 2B	− 2.24
NM_001767.2	CD2	CD2 antigen	− 2.14
NM_000565.2	IL6R	Interleukin 6 receptor	2.05
NM_156038.2	CSF3R	Colony-stimulating factor 3 receptor (granulocyte)	2.09
NM_000574.2	CD55	Decay accelerating factor	2.60
NM_000902.3	MME	Neprilysin	3.01
NM_004633.3	IL1R2	Interleukin 1 receptor, type II	3.39
NM_173343.1	IL1R2	Interleukin 1 receptor, type II	4.34
Leukocyte transendothelial migration (9)			
NM_002649.2	PIK3CG	Phosphatidylinositol-4,5-bisphosphate 3-kinase	2.01
NM_003370.3	VASP	Vasodilator-stimulated phosphoprotein	2.09
NM_000433.2	NCF2	Neutrophil cytosolic factor 2	2.11
NM_006496.1	GNAI3	Guanine nucleotide binding protein (G protein), alpha	2.11
NM_013416.2	NCF4	Neutrophil cytosolic factor 4	2.19
NM_000631.3	NCF4	Neutrophil cytosolic factor 4	2.33
NM_002859.1	PXN	Paxillin	2.49
NM_001315.1	MAPK14	p38 MAP kinase	2.87

(*Continued*)

GenBank ID	Gene symbol	Descriptive name	Fold change
NM_004994.2	MMP9	Matrix metalloproteinase-9 (gelatinase B)	5.25
Natural killer cell mediated cytotoxicity (9)			
NM_016382.2	CD244	CD244 natural killer cell receptor 2B4	− 3.24
NM_147130.1	NCR3	Natural cytotoxicity triggering receptor 3	− 2.51
NM_002351.2	SH2D1A	SH2 domain protein 1A	− 2.19
NM_007360.1	KLRK1	Killer cell lectin-like receptor subfamily K, member 1	− 2.19
NM_005041.4	PRF1	Perforin 1 (pore forming protein)	− 2.17
NM_001079.3	ZAP70	Zeta-chain (TCR) associated protein kinase	− 2.13
NM_000734.2	CD247	CD3Z antigen, zeta polypeptide	− 2.03
NM_002649.2	PIK3CG	Phosphatidylinositol-4, 5-bisphosphate 3-kinase	2.01
NM_002117.4	HLA-C	Major histocompatibility complex, class I	2.18

Supplementary Table S4. The selected gene lists evaluated by hypergeometric distribution testing based on Gene Ontology (GO) annotations to determine their component within the cells

Supplementary Table S5. The ROC curve analysis of the plasma proteins encoded by the 10 candidate genes and STEMI standard marker, CK-MB and TnT

Variable	AUC	95% CI	*P* value (vs CK-MB)	*P* value (vs TNT)
CKMB	0.587	0.465 to 0.702	–	< 0.0001
TNT	0.845	0.740 to 0.919	< 0.0001	–
CA4	0.753	0.637 to 0.847	0.0113	0.1991
CAMP	0.684	0.563 to 0.788	0.1584	0.0314
IRAK3	0.966	0.894 to 0.994	< 0.0001	0.0023
MANSC1	0.81	0.700 to 0.893	0.0002	0.5812
MMP9	0.827	0.720 to 0.906	< 0.0001	0.7666
MOSC1	0.737	0.620 to 0.834	0.0155	0.108
QPCT	0.81	0.700 to 0.893	< 0.0001	0.5514
PGLYRP1	0.944	0.863 to 0.985	< 0.0001	0.0244
TREM	0.609	0.487 to 0.722	0.6824	0.0002
VNN3	0.928	0.842 to 0.975	< 0.0001	0.0437
