# Supplementary material for: Assessment and diagnostic relevance of novel serum biomarkers for early decision of ST-elevation myocardial infarction
Source: Oncotarget. 2015 May 18;6(15):12970–83. doi: 10.18632/oncotarget.4001 (PMC4536992; doi:10.18632/oncotarget.4001)
Supplement: Supplementary file 2 [file oncotarget-06-12970-s002.docx]

**supplemental Table 2. 546 Differentially expressed genes in STEMI compared with non-STEMI group (Normal and r_STEMI)**

| Gene name | Gene Symbol | GenBank Accession | Fold Change |
| --- | --- | --- | --- |
| Arginase, liver | ARG1 | NM_000045.2 | 7.2 |
| Interleukin 18 receptor 1 | IL18R1 | NM_003855.2 | 7.2 |
| Enoyl Coenzyme A hydratase domain containing 3 | ECHDC3 | NM_024693.3 | 6.1 |
| Orosomucoid 1 | ORM1 | NM_000607.1 | 5.7 |
| Solute carrier family 11 (proton-coupled divalent metal ion transporters), member 1 | SLC11A1 | NM_000578.3 | 5.5 |
| Acyl-CoA synthetase long-chain family member 1 | ACSL1 | NM_001995.2 | 5.4 |
| C-type lectin domain family 4, member D | CLEC4D | NM_080387.4 | 5.4 |
| Matrix metallopeptidase 9 (gelatinase B, 92kDa gelatinase, 92kDa type IV collagenase) | MMP9 | NM_004994.2 | 5.2 |
| Kelch-like 2, Mayven (Drosophila) | KLHL2 | NM_007246.2 | 5.1 |
| 6-phosphofructo-2-kinase/fructose-2,6-biphosphatase 2 | PFKFB2 | NM_006212.2 | 5.0 |
| C-type lectin domain family 4, member E | CLEC4E | NM_014358.2 | 4.7 |
| G protein-coupled receptor 97 | GPR97 | NM_170776.3 | 4.4 |
| Vanin 1 | VNN1 | NM_004666.1 | 4.4 |
| FK506 binding protein 5 | FKBP5 | NM_004117.2 | 4.3 |
| Interleukin 1 receptor, type II | IL1R2 | NM_173343.1 | 4.3 |
| Chromosome 19 open reading frame 59 | C19orf59 | NM_174918.2 | 4.2 |
|  |  | AL049435 | 4.2 |
| F-box and leucine-rich repeat protein 13 | FBXL13 | NM_145032.2 | 4.1 |
| Transmembrane and coiled-coil domain family 3 | TMCC3 | NM_020698.1 | 4.0 |
| Similar to hypothetical protein LOC284701 | LOC643313 | XM_933030.1 | 3.9 |
| G protein-coupled receptor 97 | GPR97 | NM_170776.3 | 3.8 |
| Triggering receptor expressed on myeloid cells 1 | TREM1 | NM_018643.2 | 3.8 |
| Insulin receptor substrate 2 | IRS2 | NM_003749.2 | 3.7 |
| Interleukin 18 receptor accessory protein | IL18RAP | NM_003853.2 | 3.6 |
| S100 calcium binding protein P | S100P | NM_005980.2 | 3.6 |
| Heat shock 70kDa protein 1A | HSPA1A | NM_005345.4 | 3.6 |
|  |  | BG055310 | 3.5 |
| RALBP1 associated Eps domain containing 2 | REPS2 | NM_004726.2 | 3.5 |
|  |  | BX098605 | 3.4 |
| Hypothetical gene supported by BC044942 | LOC441268 | NM_001013725.1 | 3.4 |
| Interleukin 1 receptor, type II | IL1R2 | NM_004633.3 | 3.4 |
| Abhydrolase domain containing 5 | ABHD5 | NM_016006.3 | 3.3 |
| Mitogen-activated protein kinase kinase kinase 2 | MAP3K2 | XM_001128799.1 | 3.3 |
| Polyhomeotic homolog 2 (Drosophila) | PHC2 | NM_004427.3 | 3.3 |
| Kelch repeat and BTB (POZ) domain containing 7 | KBTBD7 | NM_032138.4 | 3.3 |
| KIAA0319-like | KIAA0319L | NM_024874.3 | 3.3 |
| Interleukin-1 receptor-associated kinase 3 | IRAK3 | NM_007199.1 | 3.3 |
| Zinc finger and BTB domain containing 16 | ZBTB16 | NM_001018011.1 | 3.2 |
| MANSC domain containing 1 | MANSC1 | NM_018050.2 | 3.2 |
| Cyclin-dependent kinase 5, regulatory subunit 1 (p35) | CDK5R1 | NM_003885.2 | 3.2 |
| Solute carrier family 9 (sodium/hydrogen exchanger), member 8 | SLC9A8 | NM_015266.1 | 3.2 |
| Phosphatase and actin regulator 1 | PHACTR1 | NM_030948.1 | 3.2 |
|  | SUMO1P1 | NR_002189.2 | 3.2 |
|  | LOC644935 | XM_928022.1 | 3.2 |
| Acyl-CoA synthetase long-chain family member 4 | ACSL4 | NM_004458.1 | 3.2 |
| Chondroitin sulfate synthase 1 | CHSY1 | NM_014918.3 | 3.2 |
| Peptidyl arginine deiminase, type IV | PADI4 | NM_012387.1 | 3.1 |
| Ubiquitin specific peptidase 10 | USP10 | NM_005153.2 | 3.1 |
| Potassium inwardly-rectifying channel, subfamily J, member 15 | KCNJ15 | NM_170736.1 | 3.1 |
| Peptidoglycan recognition protein 1 | PGLYRP1 | NM_005091.1 | 3.1 |
| KIAA0319-like | KIAA0319L | NM_182686.1 | 3.1 |
| Leucine-rich repeat kinase 2 | LRRK2 | NM_198578.2 | 3.1 |
| Carbonic anhydrase IV | CA4 | NM_000717.2 | 3.1 |
| Kinesin family member 1B | KIF1B | NM_183416.2 | 3.1 |
| High-mobility group box 2 | HMGB2 | NM_002129.2 | 3.0 |
| Exostoses (multiple)-like 3 | EXTL3 | NM_001440.2 | 3.0 |
| Solute carrier family 6 (neurotransmitter transporter, taurine), member 6 | SLC6A6 | NM_003043.3 | 3.0 |
| Membrane metallo-endopeptidase | MME | NM_000902.3 | 3.0 |
| SP110 nuclear body protein | SP110 | NM_004510.2 | 3.0 |
| Interferon-related developmental regulator 1 | IFRD1 | NM_001550.2 | 3.0 |
| Pannexin 2 | PANX2 | NM_052839.2 | 3.0 |
| Nuclear factor, interleukin 3 regulated | NFIL3 | NM_005384.2 | 3.0 |
| Hypothetical protein LOC284757 | LOC284757 | NM_001004305.1 | 2.9 |
| Leucine-rich alpha-2-glycoprotein 1 | LRG1 | NM_052972.2 | 2.9 |
| Solute carrier family 25 (carnitine/acylcarnitine translocase), member 20 | SLC25A20 | XM_001133926.1 | 2.9 |
| Protein kinase, DNA-activated, catalytic polypeptide | PRKDC | NM_001081640.1 | 2.9 |
| Retinitis pigmentosa GTPase regulator | RPGR | NM_001023582.1 | 2.9 |
| Mitogen-activated protein kinase 14 | MAPK14 | NM_001315.1 | 2.9 |
| Toll-like receptor 4 | TLR4 | NM_138554.2 | 2.9 |
| Tumor necrosis factor, alpha-induced protein 6 | TNFAIP6 | NM_007115.2 | 2.9 |
| Transglutaminase 3 (E polypeptide, protein-glutamine-gamma-glutamyltransferase) | TGM3 | NM_003245.2 | 2.8 |
| Purinergic receptor P2Y, G-protein coupled, 13 | P2RY13 | NM_176894.1 | 2.8 |
| Chromosome 7 open reading frame 53 | C7orf53 | NM_182597.1 | 2.8 |
|  |  | BX119134 | 2.8 |
| Oncostatin M | OSM | NM_020530.3 | 2.8 |
|  |  | AL080095 | 2.8 |
| Multiple EGF-like-domains 9 | MEGF9 | NM_001080497.1 | 2.8 |
| SFRS protein kinase 1 | SRPK1 | NM_003137.3 | 2.8 |
| WD repeat and FYVE domain containing 3 | WDFY3 | NM_178583.1 | 2.8 |
| SH3-domain GRB2-like endophilin B1 | SH3GLB1 | NM_016009.3 | 2.8 |
| Dysferlin, limb girdle muscular dystrophy 2B (autosomal recessive) | DYSF | NM_003494.2 | 2.8 |
| Stromal membrane-associated GTPase-activating protein 2 | SMAP1L | NM_022733.1 | 2.7 |
|  |  | BG217842 | 2.7 |
| G protein-coupled receptor 177 | GPR177 | NM_001002292.1 | 2.7 |
| Phosphorylase, glycogen; liver (Hers disease, glycogen storage disease type VI) | PYGL | NM_002863.3 | 2.7 |
| Phosphatidylinositol 3,4,5-trisphosphate-dependent RAC exchanger 1 | PREX1 | NM_020820.3 | 2.7 |
| Matrix metallopeptidase 25 | MMP25 | NM_022468.4 | 2.7 |
| Ring finger 144B | IBRDC2 | NM_182757.2 | 2.7 |
| 5-oxoprolinase (ATP-hydrolysing) | OPLAH | NM_017570.2 | 2.7 |
| Family with sequence similarity 126, member B | FAM126B | NM_173822.2 | 2.7 |
| Hypothetical protein FLJ10357 | FLJ10357 | NM_018071.3 | 2.7 |
| PFTAIRE protein kinase 1 | PFTK1 | NM_012395.2 | 2.7 |
| 6-phosphofructo-2-kinase/fructose-2,6-biphosphatase 3 | PFKFB3 | NM_004566.2 | 2.7 |
| Glutaminyl-peptide cyclotransferase (glutaminyl cyclase) | QPCT | NM_012413.3 | 2.7 |
| Egf-like module containing, mucin-like, hormone receptor-like 3 | EMR3 | NM_032571.3 | 2.7 |
| Yip1 domain family, member 4 | YIPF4 | NM_032312.2 | 2.6 |
| Aspartic peptidase, retroviral-like 1 | SASP | NM_152792.1 | 2.6 |
| Plexin domain containing 2 | PLXDC2 | NM_032812.7 | 2.6 |
| ST6 (alpha-N-acetyl-neuraminyl-2,3-beta-galactosyl-1,3)-N-acetylgalactosaminide alpha-2,6-sialyltransferase 2 | ST6GALNAC2 | NM_006456.1 | 2.6 |
|  |  | BX096301 | 2.6 |
| Coagulation factor V (proaccelerin, labile factor) | F5 | NM_000130.4 | 2.6 |
| Mediterranean fever | MEFV | NM_000243.1 | 2.6 |
| Teashirt zinc finger homeobox 3 | TSHZ3 | NM_020856.2 | 2.6 |
| Keratin 23 (histone deacetylase inducible) | KRT23 | NM_015515.3 | 2.6 |
| Ectonucleoside triphosphate diphosphohydrolase 1 | ENTPD1 | NM_001098175.1 | 2.6 |
| Netrin G2 | NTNG2 | NM_032536.1 | 2.6 |
|  | LOC347376 | XM_937928.1 | 2.6 |
| Transaldolase 1 | TALDO1 | XM_938697.1 | 2.6 |
| NLR family, pyrin domain containing 12 | NLRP12 | NM_144687.1 | 2.6 |
|  | NUDT16P | NR_002949.1 | 2.6 |
|  |  | AW376320 | 2.6 |
| Folate receptor 3 (gamma) | FOLR3 | NM_000804.2 | 2.6 |
| Histone cluster 1, H2bc | HIST1H2BC | NM_003526.2 | 2.6 |
| CD55 molecule, decay accelerating factor for complement (Cromer blood group) | CD55 | NM_000574.2 | 2.6 |
|  |  | AK024852 | 2.6 |
| Ubiquitin domain containing 1 | UBTD1 | NM_024954.3 | 2.6 |
| Zinc finger protein 36, C3H type-like 1 | ZFP36L1 | NM_004926.2 | 2.6 |
| Hypothetical LOC728417 | LOC728417 | XM_001130364.1 | 2.6 |
| Ropporin 1-like | ROPN1L | NM_031916.2 | 2.6 |
| KIAA0319-like | KIAA0319L | NM_024874.3 | 2.6 |
| Jumonji domain containing 2B | JMJD2B | NM_015015.1 | 2.6 |
|  | LOC153561 | NM_207331.2 | 2.6 |
| Acidic (leucine-rich) nuclear phosphoprotein 32 family, member A | ANP32A | NM_006305.2 | 2.6 |
|  | LOC731865 | XM_001134020.1 | 2.6 |
| Dehydrogenase/reductase (SDR family) member 13 | DHRS13 | NM_144683.3 | 2.6 |
| Carboxypeptidase D | CPD | NM_001304.3 | 2.6 |
|  | LOC731003 | XM_001131606.1 | 2.6 |
| Tubulin, alpha 4a | TUBA4A | NM_006000.1 | 2.6 |
| Vanin 3 | VNN3 | NM_001024460.1 | 2.6 |
| Zinc finger, FYVE domain containing 16 | ZFYVE16 | NM_014733.2 | 2.6 |
| NAD(P)H dehydrogenase, quinone 2 | NQO2 | NM_000904.2 | 2.6 |
| Vanin 2 | VNN2 | NM_004665.2 | 2.5 |
|  | LOC653157 | XM_926258.1 | 2.5 |
| Chromosome 13 open reading frame 18 | C13orf18 | NM_025113.1 | 2.5 |
| Cytoplasmic polyadenylation element binding protein 4 | CPEB4 | NM_030627.1 | 2.5 |
| Glucan (1,4-alpha-), branching enzyme 1 (glycogen branching enzyme, Andersen disease, glycogen storage disease type IV) | GBE1 | NM_000158.2 | 2.5 |
| Transmembrane protein 184B | TMEM184B | NM_012264.3 | 2.5 |
| Cytidine deaminase | CDA | NM_001785.2 | 2.5 |
| Nicotinamide phosphoribosyltransferase | PBEF1 | NM_005746.2 | 2.5 |
| NLR family, pyrin domain containing 12 | NLRP12 | NM_033297.1 | 2.5 |
| Solute carrier family 22 (organic cation/ergothioneine transporter), member 4 | SLC22A4 | NM_003059.2 | 2.5 |
| Lipid phosphate phosphatase-related protein type 2 | LPPR2 | NM_022737.1 | 2.5 |
| Brain abundant, membrane attached signal protein 1 | BASP1 | NM_006317.3 | 2.5 |
| RAB11 family interacting protein 1 (class I) | RAB11FIP1 | NM_001002814.1 | 2.5 |
| Sulfatase 2 | SULF2 | NM_018837.2 | 2.5 |
| Paxillin | PXN | NM_002859.1 | 2.5 |
| TIMP metallopeptidase inhibitor 2 | TIMP2 | NM_003255.4 | 2.5 |
| Olfactory receptor, family 10, subfamily G, member 3 | OR10G3 | NM_001005465.1 | 2.5 |
| RALBP1 associated Eps domain containing 2 | REPS2 | NM_004726.2 | 2.5 |
| Dedicator of cytokinesis 5 | DOCK5 | NM_024940.6 | 2.5 |
|  | LOC650919 | XM_944417.1 | 2.5 |
| Endoplasmic reticulum-golgi intermediate compartment (ERGIC) 1 | ERGIC1 | NM_020462.1 | 2.5 |
| Diacylglycerol O-acyltransferase homolog 2 (mouse) | DGAT2 | NM_032564.2 | 2.5 |
| DKFZp761E198 protein | DKFZp761E198 | NM_138368.3 | 2.5 |
| CCAAT/enhancer binding protein (C/EBP), delta | CEBPD | NM_005195.3 | 2.5 |
| Heme binding protein 2 | HEBP2 | NM_014320.2 | 2.5 |
| Solute carrier family 11 (proton-coupled divalent metal ion transporters), member 1 | SLC11A1 | NM_000578.3 | 2.5 |
| KIAA0232 | KIAA0232 | NM_014743.2 | 2.5 |
| UDP-Gal:betaGlcNAc beta 1,4- galactosyltransferase, polypeptide 5 | B4GALT5 | NM_004776.2 | 2.5 |
|  | dJ341D10.1 | NM_001007535.1 | 2.4 |
| Transducin-like enhancer of split 3 (E(sp1) homolog, Drosophila) | TLE3 | NM_005078.1 | 2.4 |
| Leukocyte immunoglobulin-like receptor, subfamily A (with TM domain), member 5 | LILRA5 | NM_181879.1 | 2.4 |
|  |  | CD238045 | 2.4 |
| Male-specific lethal 3-like 1 (Drosophila) | MSL3L1 | NM_078629.1 | 2.4 |
| Cytochrome P450, family 4, subfamily F, polypeptide 3 | CYP4F3 | NM_000896.1 | 2.4 |
| Chromosome 3 open reading frame 62 | C3orf62 | NM_198562.1 | 2.4 |
| Rho GTPase activating protein 24 | ARHGAP24 | NM_031305.2 | 2.4 |
| Kelch-like 8 (Drosophila) | KLHL8 | NM_020803.3 | 2.4 |
| Plexin C1 | PLXNC1 | NM_005761.1 | 2.4 |
| Frequently rearranged in advanced T-cell lymphomas | FRAT1 | NM_005479.2 | 2.4 |
| CAMP responsive element binding protein 5 | CREB5 | NM_001011666.1 | 2.4 |
| Aquaporin 9 | AQP9 | NM_020980.2 | 2.4 |
| MOCO sulphurase C-terminal domain containing 1 | MOSC1 | NM_022746.2 | 2.4 |
| Hypothetical protein LOC201175 | LOC201175 | NM_174919.2 | 2.4 |
| Cystatin A (stefin A) | CSTA | NM_005213.3 | 2.4 |
| Topoisomerase I binding, arginine/serine-rich | TOPORS | NM_005802.2 | 2.4 |
|  | FTHL12 | NR_002205.1 | 2.4 |
| ATG2 autophagy related 2 homolog A (S. cerevisiae) | ATG2A | NM_015104.1 | 2.4 |
| HLA complex group 27 | HCG27 | NM_181717.2 | 2.4 |
| Membrane bound O-acyltransferase domain containing 2 | MBOAT2 | XM_001129292.1 | 2.4 |
| Hypothetical protein LOC100130229 | STK17B | NM_004226.2 | 2.4 |
| Calcium/calmodulin-dependent protein kinase kinase 2, beta | CAMKK2 | NM_153500.1 | 2.4 |
| Cytochrome b5 reductase 4 | CYB5R4 | NM_016230.3 | 2.4 |
| Supervillin | SVIL | NM_003174.3 | 2.4 |
| Transmembrane protein 55A | TMEM55A | NM_018710.1 | 2.4 |
| Similar to VAMP (vesicle-associated membrane protein)-associated protein A, 33kDa | ABHD2 | NM_152924.3 | 2.4 |
| Acid phosphatase 6, lysophosphatidic | ACP6 | NM_016361.3 | 2.4 |
| Similar to 60S ribosomal protein L21 | LOC402176 | NM_001011538.2 | 2.4 |
| Hypothetical protein LOC285550 | BST1 | NM_004334.1 | 2.4 |
| Frequently rearranged in advanced T-cell lymphomas | FRAT1 | NM_005479.2 | 2.4 |
| Solute carrier family 22, member 17 | SLC22A17 | NM_016609.3 | 2.4 |
| Neural precursor cell expressed, developmentally down-regulated 9 | NEDD9 | NM_006403.2 | 2.4 |
| STEAP family member 4 | STEAP4 | NM_024636.2 | 2.4 |
| SAM domain, SH3 domain and nuclear localization signals 1 | SAMSN1 | NM_022136.3 | 2.4 |
| Thioredoxin domain containing 13 | TXNDC13 | NM_021156.2 | 2.4 |
| Triggering receptor expressed on myeloid cells-like 2 | TREML2 | NM_024807.1 | 2.4 |
| GRB2-associated binding protein 2 | GAB2 | NM_080491.1 | 2.4 |
| Cytoskeleton-associated protein 4 | CKAP4 | NM_006825.2 | 2.4 |
| Disrupted in renal carcinoma 2 | DIRC2 | NM_032839.1 | 2.4 |
| RAS (RAD and GEM)-like GTP binding 2 | REM2 | NM_173527.2 | 2.4 |
| Survival motor neuron domain containing 1 | SMNDC1 | NM_005871.2 | 2.3 |
| Chromosome 5 open reading frame 32 | C5orf32 | NM_032412.3 | 2.3 |
| Alkaline phosphatase, liver/bone/kidney | ALPL | NM_000478.3 | 2.3 |
| Myotubularin related protein 3 | MTMR3 | NM_021090.3 | 2.3 |
| Ring finger protein 24 | RNF24 | NM_007219.2 | 2.3 |
| Sialic acid binding Ig-like lectin 5 | SIGLEC5 | NM_003830.1 | 2.3 |
| Formyl peptide receptor 2 | FPRL1 | NM_001005738.1 | 2.3 |
| Neutrophil cytosolic factor 4, 40kDa | NCF4 | NM_000631.3 | 2.3 |
| Cysteine sulfinic acid decarboxylase | CSAD | NM_015989.3 | 2.3 |
| 6-phosphofructo-2-kinase/fructose-2,6-biphosphatase 4 | PFKFB4 | NM_004567.2 | 2.3 |
| Regulator of G-protein signaling 2, 24kDa | RGS2 | NM_002923.2 | 2.3 |
| Protein phosphatase 1D magnesium-dependent, delta isoform | PPM1D | NM_003620.2 | 2.3 |
| Synaptotagmin-like 3 | SYTL3 | NM_001009991.2 | 2.3 |
| Hypothetical LOC728054 | LOC728054 | XM_001128749.1 | 2.3 |
| CAMP responsive element binding protein 5 | CREB5 | NM_182898.2 | 2.3 |
| IKK interacting protein | IKIP | NM_153687.2 | 2.3 |
| Regulatory factor X, 2 (influences HLA class II expression) | RFX2 | NM_000635.2 | 2.3 |
| WD repeat and SOCS box-containing 1 | WSB1 | NM_134265.2 | 2.3 |
| Bestrophin 1 | BEST1 | NM_004183.2 | 2.3 |
| B-cell CLL/lymphoma 3 | BCL3 | NM_005178.3 | 2.3 |
| Adenylate cyclase 4 | ADCY4 | NM_139247.2 | 2.3 |
|  |  | AK129555 | 2.3 |
| Mannosidase, alpha, class 2A, member 2 | MAN2A2 | NM_006122.2 | 2.3 |
|  |  | BI821208 | 2.3 |
| Death-associated protein kinase 2 | DAPK2 | NM_014326.3 | 2.3 |
| S100 calcium binding protein A11 | S100A11 | NM_005620.1 | 2.3 |
| ER lipid raft associated 1 | ERLIN1 | NM_006459.2 | 2.3 |
|  |  | BC035098 | 2.3 |
| Hypothetical gene supported by AK093729; BX647918 | LOC441124 | XM_499022.3 | 2.3 |
| V-ets erythroblastosis virus E26 oncogene homolog 2 (avian) | ETS2 | NM_005239.4 | 2.3 |
| Cat eye syndrome chromosome region, candidate 6 | CECR6 | NM_031890.2 | 2.3 |
| Arachidonate 5-lipoxygenase-activating protein | ALOX5AP | NM_001629.2 | 2.3 |
|  |  | BX111162 | 2.3 |
| Absent in melanoma 2 | AIM2 | NM_004833.1 | 2.3 |
| C-type lectin domain family 7, member A | CLEC7A | NM_197948.2 | 2.3 |
| Src-like-adaptor | SLA | NM_006748.1 | 2.3 |
| Histidine ammonia-lyase | HAL | NM_002108.2 | 2.3 |
| Signal-regulatory protein beta 1 | SIRPB1 | NM_006065.1 | 2.3 |
| NLR family, CARD domain containing 4 | NLRC4 | NM_021209.3 | 2.3 |
| B-cell CLL/lymphoma 6 (zinc finger protein 51) | BCL6 | NM_001706.2 | 2.3 |
| Proline-serine-threonine phosphatase interacting protein 2 | PSTPIP2 | NM_024430.2 | 2.2 |
| MAX dimerization protein 3 | MXD3 | NM_031300.2 | 2.2 |
| Zinc finger protein 438 | ZNF438 | NM_182755.1 | 2.2 |
| PHD finger protein 12 | PHF12 | NM_001033561.1 | 2.2 |
| Superoxide dismutase 2, mitochondrial | SOD2 | NM_000636.2 | 2.2 |
| Interferon, gamma-inducible protein 16 | IFI16 | NM_005531.2 | 2.2 |
| 1-acylglycerol-3-phosphate O-acyltransferase 9 | MAG1 | NM_032717.3 | 2.2 |
| Transmembrane protein 120A | TMEM120A | NM_031925.1 | 2.2 |
| Carcinoembryonic antigen-related cell adhesion molecule 3 | CEACAM3 | NM_001815.2 | 2.2 |
| Ecotropic viral integration site 2A | EVI2A | NM_014210.2 | 2.2 |
|  |  | BX111393 | 2.2 |
| Chromosome 9 open reading frame 72 | C9orf72 | NM_018325.1 | 2.2 |
|  |  | DA674418 | 2.2 |
| Protein phosphatase 1, regulatory (inhibitor) subunit 3B | PPP1R3B | NM_024607.2 | 2.2 |
| Retinol binding protein 7, cellular | RBP7 | NM_052960.1 | 2.2 |
|  |  | AW273831 | 2.2 |
| OTU domain containing 1 | OTUD1 | XM_001134465.1 | 2.2 |
|  |  | AK092074 | 2.2 |
| H2.0-like homeobox | HLX | NM_021958.2 | 2.2 |
| Sorting nexin 13 | SNX13 | NM_015132.3 | 2.2 |
| Myelin protein zero-like 2 | EVA1 | NM_005797.2 | 2.2 |
| ATP-binding cassette, sub-family G (WHITE), member 1 | ABCG1 | NM_016818.2 | 2.2 |
| Strawberry notch homolog 2 (Drosophila) | SBNO2 | NM_014963.2 | 2.2 |
| Glycerol kinase | GK | NM_000167.3 | 2.2 |
| Cysteine-rich secretory protein LCCL domain containing 2 | CRISPLD2 | NM_031476.2 | 2.2 |
| LON peptidase N-terminal domain and ring finger 1 | LONRF1 | NM_152271.3 | 2.2 |
| Putative homeodomain transcription factor 1 | PHTF1 | NM_006608.1 | 2.2 |
| Complement component 1, r subcomponent-like | C1RL | NM_016546.1 | 2.2 |
| Neutrophil cytosolic factor 4, 40kDa | NCF4 | NM_013416.2 | 2.2 |
|  |  | AV705309 | 2.2 |
| Casein kinase 1, delta | CSNK1D | NM_139062.1 | 2.2 |
| IQ motif containing GTPase activating protein 1 | IQGAP1 | NM_003870.3 | 2.2 |
|  |  | AW975939 | 2.2 |
| Major histocompatibility complex, class I, C | HLA-C | NM_002117.4 | 2.2 |
| Chromosome 20 open reading frame 3 | C20orf3 | NM_020531.2 | 2.2 |
| Caspase 5, apoptosis-related cysteine peptidase | CASP5 | NM_004347.1 | 2.2 |
| Solute carrier family 16, member 3 (monocarboxylic acid transporter 4) | SLC16A3 | NM_004207.2 | 2.2 |
| Thioredoxin domain containing 3 (spermatozoa) | TXNDC3 | NM_016616.3 | 2.2 |
| NFAT activating protein with ITAM motif 1 | NFAM1 | NM_145912.5 | 2.2 |
|  |  | BM716742 | 2.2 |
| Reticulocalbin 3, EF-hand calcium binding domain | RCN3 | NM_020650.2 | 2.2 |
| Mitogen-activated protein kinase kinase kinase kinase 4 | MAP4K4 | NM_145686.2 | 2.2 |
| Protein phosphatase 4, regulatory subunit 1 | PPP4R1 | NM_005134.2 | 2.2 |
| Platelet-activating factor receptor | PTAFR | NM_000952.3 | 2.2 |
| Chromosome 10 open reading frame 73 | C10orf73 | XM_096317.11 | 2.2 |
| Syntaxin binding protein 5 (tomosyn) | STXBP5 | NM_139244.2 | 2.2 |
| Solute carrier family 12 (potassium/chloride transporters), member 6 | SLC12A6 | NM_001042496.1 | 2.2 |
| Prostaglandin-endoperoxide synthase 2 (prostaglandin G/H synthase and cyclooxygenase) | PTGS2 | NM_000963.1 | 2.2 |
| KIAA1324 | KIAA1324 | NM_020775.2 | 2.2 |
| CKLF-like MARVEL transmembrane domain containing 2 | CMTM2 | NM_144673.2 | 2.2 |
|  | LOC642678 | XM_926130.1 | 2.2 |
| Zinc finger protein 281 | ZNF281 | NM_012482.3 | 2.2 |
|  |  | AK098095 | 2.2 |
| Interleukin 17 receptor A | IL17RA | NM_014339.4 | 2.2 |
| Chondroitin sulfate N-acetylgalactosaminyltransferase 2 | GALNACT-2 | NM_018590.3 | 2.1 |
| Formyl peptide receptor 1 | FPR1 | NM_002029.3 | 2.1 |
| Insulin-like growth factor 2 receptor | IGF2R | NM_000876.2 | 2.1 |
| Protein phosphatase 1, regulatory (inhibitor) subunit 3D | PPP1R3D | NM_006242.3 | 2.1 |
| Nucleotide-binding oligomerization domain containing 2 | NOD2 | NM_022162.1 | 2.1 |
| MAP kinase interacting serine/threonine kinase 1 | MKNK1 | NM_003684.3 | 2.1 |
| Quaking homolog, KH domain RNA binding (mouse) | QKI | NM_006775.1 | 2.1 |
| Phosphatidylinositol binding clathrin assembly protein | PICALM | NM_007166.2 | 2.1 |
|  | LOC648852 | XM_940430.1 | 2.1 |
| Syntaxin 3 | STX3 | NM_004177.3 | 2.1 |
| Guanine nucleotide binding protein (G protein), q polypeptide | GNAQ | NM_002072.2 | 2.1 |
|  |  | AW440143 | 2.1 |
|  |  | BX092531 | 2.1 |
|  | LOC648998 | XM_938078.2 | 2.1 |
| Ets2 repressor factor | ERF | NM_006494.1 | 2.1 |
|  |  | AA179392 | 2.1 |
| Chemokine (C-X-C motif) ligand 16 | CXCL16 | NM_022059.1 | 2.1 |
| Pecanex homolog (Drosophila) | PCNX | NM_014982.2 | 2.1 |
| Kinesin family member 1B | KIF1B | NM_015074.2 | 2.1 |
| C-type lectin domain family 5, member A | CLEC5A | NM_013252.2 | 2.1 |
| Zinc finger protein 516 | ZNF516 | XM_496278.3 | 2.1 |
| Male-specific lethal 3-like 1 (Drosophila) | MSL3L1 | NM_078628.1 | 2.1 |
| Solute carrier family 22, member 15 | SLC22A15 | NM_018420.1 | 2.1 |
| Hypothetical protein LOC56755 | SAP30L | NM_024632.4 | 2.1 |
| Membrane bound O-acyltransferase domain containing 7 | LENG4 | NM_024298.2 | 2.1 |
| FLJ42957 protein | FLJ42957 | NM_207436.1 | 2.1 |
| Acyl-Coenzyme A oxidase 1, palmitoyl | ACOX1 | NM_007292.4 | 2.1 |
| Family with sequence similarity 101, member B | FAM101B | NM_182705.2 | 2.1 |
| Fc fragment of IgG, low affinity IIa, receptor (CD32) | FCGR2A | NM_021642.2 | 2.1 |
| Hypothetical protein FLJ22662 | FLJ22662 | NM_024829.5 | 2.1 |
| Zinc finger CCCH-type, antiviral 1 | ZC3HAV1 | NM_020119.3 | 2.1 |
| Guanine nucleotide binding protein (G protein), alpha inhibiting activity polypeptide 3 | GNAI3 | NM_006496.1 | 2.1 |
| Prokineticin 2 | PROK2 | NM_021935.2 | 2.1 |
| Leukocyte immunoglobulin-like receptor, subfamily B (with TM and ITIM domains), member 3 | LILRB3 | NM_006864.2 | 2.1 |
| Neutrophil cytosolic factor 2 (65kDa, chronic granulomatous disease, autosomal 2) | NCF2 | NM_000433.2 | 2.1 |
| Endoplasmic reticulum-golgi intermediate compartment (ERGIC) 1 | ERGIC1 | NM_001031711.1 | 2.1 |
| 5'-nucleotidase, cytosolic III | NT5C3 | NM_001002010.1 | 2.1 |
| CBF1 interacting corepressor | CIR | NM_004882.3 | 2.1 |
|  |  | BX110921 | 2.1 |
| Dipeptidase 3 | DPEP3 | NM_022357.1 | 2.1 |
| MAX dimerization protein 1 | MXD1 | NM_002357.2 | 2.1 |
| Eukaryotic translation initiation factor 2C, 4 | EIF2C4 | NM_017629.2 | 2.1 |
| Egl nine homolog 1 (C. elegans) | EGLN1 | NM_022051.1 | 2.1 |
| F-box and leucine-rich repeat protein 5 | FBXL5 | NM_012161.2 | 2.1 |
| Vasodilator-stimulated phosphoprotein | VASP | NM_003370.3 | 2.1 |
| Colony stimulating factor 3 receptor (granulocyte) | CSF3R | NM_156038.2 | 2.1 |
| Recombination signal binding protein for immunoglobulin kappa J region | RBPJ | NM_203284.1 | 2.1 |
|  |  | AK123264 | 2.1 |
| Myosin binding protein C, cardiac | MYBPC3 | NM_000256.3 | 2.1 |
| Ubiquitin specific peptidase 15 | USP15 | NM_006313.1 | 2.1 |
| Neurobeachin-like 2 | NBEAL2 | NM_015175.1 | 2.1 |
|  | LOC730740 | XM_001128558.1 | 2.1 |
| Ral guanine nucleotide dissociation stimulator-like 4 | Rgr | NM_153615.1 | 2.1 |
| Coagulation factor II (thrombin) receptor-like 1 | F2RL1 | NM_005242.3 | 2.1 |
| Exportin 6 | XPO6 | NM_015171.2 | 2.1 |
| Myelin protein zero-like 1 | MPZL1 | NM_003953.4 | 2.1 |
| Cholinergic receptor, nicotinic, alpha 10 | CHRNA10 | NM_020402.2 | 2.1 |
| Hematological and neurological expressed 1 | HN1 | NM_001002033.1 | 2.1 |
| Sortilin-related receptor, L(DLR class) A repeats-containing | SORL1 | NM_003105.3 | 2.1 |
| Syntaxin 7 | STX7 | NM_003569.1 | 2.1 |
|  | LOC651738 | XM_944898.2 | 2.1 |
| Pyruvate dehydrogenase kinase, isozyme 3 | PDK3 | NM_005391.2 | 2.1 |
| Chitobiase, di-N-acetyl- | CTBS | NM_004388.1 | 2.1 |
| GABA(A) receptor-associated protein like 1 | GABARAPL1 | NM_031412.2 | 2.1 |
|  | SUMO1P3 | NR_002190.1 | 2.1 |
| Zinc finger CCCH-type, antiviral 1 | ZC3HAV1 | NM_024625.3 | 2.1 |
| Transmembrane protein 16K | TMEM16K | NM_018075.3 | 2.1 |
| Ankyrin repeat and BTB (POZ) domain containing 1 | ABTB1 | NM_032548.2 | 2.1 |
|  | LOC652616 | XM_942152.1 | 2.1 |
| Anterior pharynx defective 1 homolog B (C. elegans) | APH1B | NM_031301.2 | 2.1 |
| ST3 beta-galactoside alpha-2,3-sialyltransferase 4 | ST3GAL4 | NM_006278.1 | 2.0 |
| Rho GTPase activating protein 19 | ARHGAP19 | NM_032900.4 | 2.0 |
| Purinergic receptor P2X, ligand-gated ion channel, 1 | P2RX1 | NM_002558.2 | 2.0 |
| Interleukin 6 receptor | IL6R | NM_000565.2 | 2.0 |
| Solute carrier family 31 (copper transporters), member 2 | SLC31A2 | NM_001860.2 | 2.0 |
|  |  | AV649053 | 2.0 |
| Nicotinamide phosphoribosyltransferase | PBEF1 | NM_005746.2 | 2.0 |
|  | LOC729021 | XR_015834.1 | 2.0 |
| Hypothetical LOC399744 | LOC399744 | XM_001133357.1 | 2.0 |
| Abhydrolase domain containing 4 | ABHD4 | NM_022060.2 | 2.0 |
| Solute carrier family 2 (facilitated glucose transporter), member 3 | SLC2A3 | NM_006931.1 | 2.0 |
| Influenza virus NS1A binding protein | IVNS1ABP | NM_006469.4 | 2.0 |
| ELOVL family member 5, elongation of long chain fatty acids (FEN1/Elo2, SUR4/Elo3-like, yeast) | ELOVL5 | NM_021814.3 | 2.0 |
| Fucosyltransferase 7 (alpha (1,3) fucosyltransferase) | FUT7 | NM_004479.2 | 2.0 |
| Zinc finger protein 467 | ZNF467 | NM_207336.1 | 2.0 |
| Annexin A11 | ANXA11 | NM_145869.1 | 2.0 |
| NLR family member X1 | NLRX1 | NM_170722.1 | 2.0 |
| P450 (cytochrome) oxidoreductase | POR | NM_000941.2 | 2.0 |
| Family with sequence similarity 49, member A | FAM49A | NM_030797.2 | 2.0 |
| Mitochondrial methionyl-tRNA formyltransferase | MTFMT | NM_139242.2 | 2.0 |
| RAS guanyl releasing protein 4 | RASGRP4 | NM_170604.2 | 2.0 |
| Serine incorporator 1 | SERINC1 | NM_020755.2 | 2.0 |
| Neuroplastin | NPTN | NM_012428.2 | 2.0 |
| Ribonucleotide reductase M2 B (TP53 inducible) | RRM2B | NM_015713.3 | 2.0 |
| Hexokinase 2 | HK2 | NM_000189.4 | 2.0 |
| Eukaryotic translation initiation factor 2-alpha kinase 2 | EIF2AK2 | NM_002759.1 | 2.0 |
| Chromosome 1 open reading frame 91 | C1orf91 | NM_019118.2 | 2.0 |
|  |  | AK124771 | 2.0 |
| Phosphoinositide-3-kinase, catalytic, gamma polypeptide | PIK3CG | NM_002649.2 | 2.0 |
|  | FTHL2 | NR_002200.1 | 2.0 |
| Nudix (nucleoside diphosphate linked moiety X)-type motif 16 | NUDT16 | NM_152395.1 | 2.0 |
| Myosin IF | MYO1F | NM_012335.2 | 2.0 |
| WAS/WASL interacting protein family, member 1 | WIPF1 | NM_003387.4 | 2.0 |
| Multiple C2 domains, transmembrane 2 | MCTP2 | NM_018349.2 | 2.0 |
| Cathelicidin antimicrobial peptide | CAMP | NM_004345.3 | 2.0 |
|  |  | CA396207 | 2.0 |
| ATG16 autophagy related 16-like 2 (S. cerevisiae) | ATG16L2 | NM_033388.1 | 2.0 |
| Hypothetical protein KIAA1434 | KIAA1434 | NM_019593.3 | 2.0 |
| PDZ and LIM domain 7 (enigma) | PDLIM7 | NM_213636.1 | 2.0 |
| Transmembrane 6 superfamily member 1 | TM6SF1 | NM_023003.2 | 2.0 |
| B9 protein domain 2 | MGC4093 | NM_030578.2 | 2.0 |
| NLR family, pyrin domain containing 12 | NLRP12 | NM_144687.1 | 2.0 |
| Cytochrome P450, family 1, subfamily B, polypeptide 1 | CYP1B1 | NM_000104.2 | 2.0 |
| Interleukin 8 receptor, alpha | IL8RA | NM_000634.2 | 2.0 |
| Zinc finger, DHHC-type containing 18 | ZDHHC18 | NM_032283.1 | 2.0 |
| LYR motif containing 4 | LYRM4 | NM_020408.3 | -2.0 |
| Phosphoprotein enriched in astrocytes 15 | PEA15 | NM_003768.2 | -2.0 |
| Pyrin and HIN domain family, member 1 | PYHIN1 | NM_198930.2 | -2.0 |
| Cysteine-rich protein 1 (intestinal) | CRIP1 | NM_001311.3 | -2.0 |
| Signaling lymphocytic activation molecule family member 1 | SLAMF1 | NM_003037.1 | -2.0 |
| Chromosome 10 open reading frame 38 | C10orf38 | NM_001010924.1 | -2.0 |
| Hermansky-Pudlak syndrome 1 | HPS1 | NM_182639.1 | -2.0 |
| Lectin, galactoside-binding, soluble, 3 binding protein | LGALS3BP | NM_005567.2 | -2.0 |
| RAB11 family interacting protein 5 (class I) | RAB11FIP5 | NM_015470.2 | -2.0 |
| Retinoic acid receptor responder (tazarotene induced) 3 | RARRES3 | NM_004585.3 | -2.0 |
| Single immunoglobulin and toll-interleukin 1 receptor (TIR) domain | SIGIRR | NM_021805.1 | -2.0 |
| CD247 molecule | CD247 | NM_000734.2 | -2.0 |
| ORM1-like 3 (S. cerevisiae) | ORMDL3 | NM_139280.1 | -2.0 |
| Family with sequence similarity 84, member B | FAM84B | NM_174911.3 | -2.0 |
| Growth factor independent 1B transcription repressor | GFI1B | NM_004188.3 | -2.0 |
| B-cell CLL/lymphoma 11B (zinc finger protein) | BCL11B | NM_138576.2 | -2.0 |
| Hermansky-Pudlak syndrome 1 | HPS1 | NM_000195.2 | -2.0 |
| Protor-2 | FLJ14213 | NM_024841.3 | -2.0 |
| Guanosine monophosphate reductase | GMPR | NM_006877.2 | -2.0 |
| Dual specificity phosphatase 5 | DUSP5 | NM_004419.3 | -2.0 |
|  | LOC387841 | XM_932678.1 | -2.0 |
| Mitogen-activated protein kinase kinase kinase kinase 1 | MAP4K1 | NM_001042600.1 | -2.0 |
| Hydroxysteroid (17-beta) dehydrogenase 8 | HSD17B8 | NM_014234.3 | -2.0 |
| Solute carrier family 2 (facilitated glucose transporter), member 6 | SLC2A6 | NM_017585.2 | -2.0 |
|  | LOC648470 | XM_937514.1 | -2.0 |
| RAP2A, member of RAS oncogene family | RAP2A | NM_021033.5 | -2.0 |
| CD96 molecule | CD96 | NM_198196.2 | -2.0 |
| Sterile alpha motif domain containing 3 | SAMD3 | NM_152552.2 | -2.1 |
|  | LOC388275 | XM_928429.1 | -2.1 |
| Chromosome 20 open reading frame 27 | C20orf27 | NM_001039140.1 | -2.1 |
| Related RAS viral (r-ras) oncogene homolog | RRAS | NM_006270.3 | -2.1 |
| Leucine rich repeat containing 33 | LRRC33 | NM_198565.1 | -2.1 |
| Cell division cycle 25 homolog B (S. pombe) | CDC25B | NM_021872.2 | -2.1 |
| Serine threonine kinase 39 (STE20/SPS1 homolog, yeast) | STK39 | NM_013233.2 | -2.1 |
| Ral guanine nucleotide dissociation stimulator | RALGDS | NM_006266.2 | -2.1 |
| Peroxiredoxin 2 | PRDX2 | NM_181738.1 | -2.1 |
|  |  | AL832164 | -2.1 |
| Transcription elongation factor A (SII), 3 | TCEA3 | NM_003196.1 | -2.1 |
| Axin 2 (conductin, axil) | AXIN2 | NM_004655.2 | -2.1 |
| Heat shock 10kDa protein 1 (chaperonin 10) | HSPE1 | NM_002157.1 | -2.1 |
| Similar to HSPC323 | LOC284422 | XM_209196.5 | -2.1 |
| AHNAK nucleoprotein | AHNAK | NM_001620.1 | -2.1 |
| Major histocompatibility complex, class II, DP beta 1 | HLA-DPB1 | NM_002121.4 | -2.1 |
| Solute carrier family 2, (facilitated glucose transporter) member 8 | SLC2A8 | NM_014580.3 | -2.1 |
| Family with sequence similarity 38, member A | FAM38A | NM_014745.1 | -2.1 |
| Aminolevulinate, delta-, synthase 2 | ALAS2 | NM_001037967.1 | -2.1 |
| G protein-coupled receptor 68 | GPR68 | NM_003485.3 | -2.1 |
| Major histocompatibility complex, class II, DQ alpha 1 | HLA-DQA1 | XM_936128.2 | -2.1 |
| Sterile alpha motif domain containing 14 | SAMD14 | NM_174920.2 | -2.1 |
| Enah/Vasp-like | EVL | NM_016337.2 | -2.1 |
| Kruppel-like factor 12 | KLF12 | NM_007249.4 | -2.1 |
| Vasohibin 1 | VASH1 | NM_014909.3 | -2.1 |
| Zeta-chain (TCR) associated protein kinase 70kDa | ZAP70 | NM_001079.3 | -2.1 |
| Protein tyrosine phosphatase, non-receptor type 4 (megakaryocyte) | PTPN4 | NM_002830.2 | -2.1 |
| Granulysin | GNLY | NM_006433.2 | -2.1 |
| Family with sequence similarity 113, member B | FAM113B | NM_138371.1 | -2.1 |
| Chemokine (C-X-C motif) receptor 3 | CXCR3 | NM_001504.1 | -2.1 |
| CD2 molecule | CD2 | NM_001767.2 | -2.1 |
| G protein-coupled receptor 18 | GPR18 | NM_001098200.1 | -2.1 |
|  | HNRPA1P4 | XM_939887.2 | -2.1 |
| Endoplasmic reticulum aminopeptidase 2 | LRAP | NM_022350.2 | -2.1 |
| Perforin 1 (pore forming protein) | PRF1 | NM_005041.4 | -2.2 |
| Chemokine (C-C motif) ligand 5 | CCL5 | NM_002985.2 | -2.2 |
| Granulysin | GNLY | NM_012483.1 | -2.2 |
|  | LOC652775 | XM_942419.1 | -2.2 |
| Killer cell lectin-like receptor subfamily C, member 4 | KLRK1 | NM_007360.1 | -2.2 |
| SH2 domain protein 1A, Duncan's disease (lymphoproliferative syndrome) | SH2D1A | NM_002351.2 | -2.2 |
| Oligodendrocyte transcription factor 1 | OLIG1 | NM_138983.1 | -2.2 |
|  | CCL4L2 | NM_207007.2 | -2.2 |
| KIAA0020 | KIAA0020 | NM_014878.4 | -2.2 |
| Overexpressed in colon carcinoma-1 | LOC387882 | NM_207376.1 | -2.2 |
| Fatty acid synthase | FASN | NM_004104.4 | -2.2 |
| Carbonic anhydrase I | CA1 | NM_001738.1 | -2.2 |
| Fc receptor-like 3 | FCRL3 | NM_001024667.1 | -2.2 |
| Ectonucleotide pyrophosphatase/phosphodiesterase 4 (putative function) | ENPP4 | NM_014936.3 | -2.2 |
| Tropomodulin 1 | TMOD1 | NM_003275.2 | -2.2 |
| SH3-domain kinase binding protein 1 | SH3KBP1 | NM_001024666.1 | -2.2 |
| Integrin, alpha 2b (platelet glycoprotein IIb of IIb/IIIa complex, antigen CD41) | ITGA2B | NM_000419.3 | -2.2 |
| Fc fragment of IgE, high affinity I, receptor for; alpha polypeptide | FCER1A | NM_002001.2 | -2.2 |
| Sterile alpha motif domain containing 3 | SAMD3 | NM_001017373.1 | -2.2 |
| Integrin, beta 7 | ITGB7 | NM_000889.1 | -2.3 |
| Hairy and enhancer of split 4 (Drosophila) | HES4 | NM_021170.2 | -2.3 |
| Chromosome 20 open reading frame 149 | C20orf149 | NM_024299.2 | -2.3 |
| Pyrin and HIN domain family, member 1 | PYHIN1 | NM_198930.2 | -2.3 |
| CD8a molecule | CD8A | NM_171827.2 | -2.3 |
| CD3g molecule, gamma (CD3-TCR complex) | CD3G | NM_000073.1 | -2.3 |
| Coiled-coil domain containing 65 | CCDC65 | NM_033124.3 | -2.3 |
| CD6 molecule | CD6 | NM_006725.2 | -2.3 |
| CD81 molecule | CD81 | NM_004356.3 | -2.3 |
| Neurocalcin delta | NCALD | NM_001040628.1 | -2.3 |
| PDZ domain containing 4 | PDZD4 | NM_032512.2 | -2.3 |
| Keratin 1 (epidermolytic hyperkeratosis) | KRT1 | NM_006121.3 | -2.3 |
| Pleckstrin homology domain containing, family F (with FYVE domain) member 1 | PLEKHF1 | NM_024310.4 | -2.3 |
|  | LOC649923 | XM_939003.1 | -2.3 |
| G protein-coupled receptor 56 | GPR56 | NM_201524.1 | -2.3 |
| Tropomyosin 2 (beta) | TPM2 | NM_213674.1 | -2.3 |
| Transforming growth factor, beta receptor III | TGFBR3 | NM_003243.2 | -2.3 |
| Solute carrier family 4, anion exchanger, member 1 (erythrocyte membrane protein band 3, Diego blood group) | SLC4A1 | NM_000342.2 | -2.3 |
| Interleukin 32 | IL32 | NM_001012636.1 | -2.3 |
| Uncoupling protein 2 (mitochondrial, proton carrier) | UCP2 | NM_003355.2 | -2.4 |
| Runt-related transcription factor 3 | RUNX3 | NM_004350.2 | -2.4 |
| Family with sequence similarity 62 (C2 domain containing), member A | FAM62A | NM_015292.1 | -2.4 |
| Lectin, galactoside-binding, soluble, 2 | LGALS2 | NM_006498.2 | -2.4 |
|  | LOC651751 | XM_940969.1 | -2.4 |
| Transmembrane protein 158 | TMEM158 | NM_015444.2 | -2.4 |
|  |  | BC070337 | -2.4 |
| SH3-binding domain kinase 1 | SBK1 | NM_001024401.2 | -2.4 |
| Carbohydrate (chondroitin 4) sulfotransferase 12 | CHST12 | NM_018641.3 | -2.4 |
| Cathepsin L-like 3 | CTSL1 | NM_001912.3 | -2.4 |
| Chemokine (C-X3-C motif) receptor 1 | CX3CR1 | NM_001337.3 | -2.4 |
| Lanosterol synthase (2,3-oxidosqualene-lanosterol cyclase) | LSS | NM_002340.3 | -2.5 |
| DNA segment on chromosome 4 (unique) 234 expressed sequence | D4S234E | NM_001040101.1 | -2.5 |
| Natural cytotoxicity triggering receptor 3 | NCR3 | NM_147130.1 | -2.5 |
|  |  | CR596519 | -2.5 |
| Interleukin 2 receptor, beta | IL2RB | NM_000878.2 | -2.5 |
| Cathepsin W | CTSW | NM_001335.3 | -2.5 |
| Fibroblast growth factor binding protein 2 | FGFBP2 | NM_031950.2 | -2.5 |
| LFNG O-fucosylpeptide 3-beta-N-acetylglucosaminyltransferase | LFNG | NM_001040167.1 | -2.6 |
| Granzyme M (lymphocyte met-ase 1) | GZMM | NM_005317.2 | -2.6 |
| Coiled-coil domain containing 102A | CCDC102A | NM_033212.2 | -2.6 |
| Killer cell lectin-like receptor subfamily G, member 1 | KLRG1 | NM_005810.3 | -2.6 |
| Adenosine deaminase | ADA | NM_000022.2 | -2.6 |
| T cell receptor gamma constant 2 | TARP | NM_001003806.1 | -2.7 |
| Zinc finger protein 683 | ZNF683 | NM_173574.2 | -2.7 |
| Calcium/calmodulin-dependent protein kinase I | CAMK1 | NM_003656.3 | -2.7 |
| Platelet factor 4 variant 1 | PF4V1 | NM_002620.2 | -2.8 |
| Lymphocyte-activation gene 3 | LAG3 | NM_002286.4 | -3.0 |
| Calcium channel, voltage-dependent, gamma subunit 6 | CACNG6 | NM_031897.2 | -3.1 |
| Natural killer cell group 7 sequence | NKG7 | NM_005601.3 | -3.1 |
| Eomesodermin homolog (Xenopus laevis) | EOMES | NM_005442.2 | -3.1 |
| Granzyme H (cathepsin G-like 2, protein h-CCPX) | GZMH | NM_033423.3 | -3.2 |
| G protein-coupled receptor 114 | GPR114 | NM_153837.1 | -3.2 |
| CD244 molecule, natural killer cell receptor 2B4 | CD244 | NM_016382.2 | -3.2 |
| Granzyme A (granzyme 1, cytotoxic T-lymphocyte-associated serine esterase 3) | GZMA | NM_006144.2 | -3.4 |
| Charcot-Leyden crystal protein | CLC | NM_001828.4 | -3.6 |
| Protein phosphatase 2 (formerly 2A), regulatory subunit B, beta isoform | PPP2R2B | NM_181676.1 | -3.7 |
| Granzyme K (granzyme 3; tryptase II) | GZMK | NM_002104.2 | -3.8 |
