# Supplementary material for: Assessment and diagnostic relevance of novel serum biomarkers for early decision of ST-elevation myocardial infarction
Source: Oncotarget. 2015 May 18;6(15):12970–83. doi: 10.18632/oncotarget.4001 (PMC4536992; doi:10.18632/oncotarget.4001)
Supplement: Supplementary file 3 [file oncotarget-06-12970-s003.docx]

**Supplemental Table 4. The selected gene lists evaluated by hypergeometric distribution testing based on Gene Ontology (GO) annotations to determine their component within the cells**

| Gene name | Gene Symbol | GenBank Accession | Fold Change |
| --- | --- | --- | --- |
| Interleukin 18 receptor 1 | IL18R1 | NM_003855.2 | 7.2 |
| Orosomucoid 1 | ORM1 | NM_000607.1 | 5.7 |
| Solute carrier family 11 (proton-coupled divalent metal ion transporters), member 1 | SLC11A1 | NM_000578.3 | 5.5 |
| Acyl-CoA synthetase long-chain family member 1 | ACSL1 | NM_001995.2 | 5.4 |
| C-type lectin domain family 4, member D | CLEC4D | NM_080387.4 | 5.4 |
| Matrix metallopeptidase 9 (gelatinase B, 92kDa gelatinase, 92kDa type IV collagenase) | MMP9 | NM_004994.2 | 5.2 |
| C-type lectin domain family 4, member E | CLEC4E | NM_014358.2 | 4.7 |
| G protein-coupled receptor 97 | GPR97 | NM_170776.3 | 4.4 |
| Interleukin 1 receptor, type II | IL1R2 | NM_173343.1 | 4.3 |
| Transmembrane and coiled-coil domain family 3 | TMCC3 | NM_020698.1 | 4.0 |
| G protein-coupled receptor 97 | GPR97 | NM_170776.3 | 3.8 |
| Triggering receptor expressed on myeloid cells 1 | TREM1 | NM_018643.2 | 3.8 |
| Interleukin 18 receptor accessory protein | IL18RAP | NM_003853.2 | 3.6 |
| Interleukin 1 receptor, type II | IL1R2 | NM_004633.3 | 3.4 |
| MANSC domain containing 1 | MANSC1 | NM_018050.2 | 3.2 |
| Solute carrier family 9 (sodium/hydrogen exchanger), member 8 | SLC9A8 | NM_015266.1 | 3.2 |
| Acyl-CoA synthetase long-chain family member 4 | ACSL4 | NM_004458.1 | 3.2 |
| Chondroitin sulfate synthase 1 | CHSY1 | NM_014918.3 | 3.2 |
| Potassium inwardly-rectifying channel, subfamily J, member 15 | KCNJ15 | NM_170736.1 | 3.1 |
| Peptidoglycan recognition protein 1 | PGLYRP1 | NM_005091.1 | 3.1 |
| Exostoses (multiple)-like 3 | EXTL3 | NM_001440.2 | 3.0 |
| Solute carrier family 6 (neurotransmitter transporter, taurine), member 6 | SLC6A6 | NM_003043.3 | 3.0 |
| Membrane metallo-endopeptidase | MME | NM_000902.3 | 3.0 |
| Pannexin 2 | PANX2 | NM_052839.2 | 3.0 |
| Leucine-rich alpha-2-glycoprotein 1 | LRG1 | NM_052972.2 | 2.9 |
| Toll-like receptor 4 | TLR4 | NM_138554.2 | 2.9 |
| Tumor necrosis factor, alpha-induced protein 6 | TNFAIP6 | NM_007115.2 | 2.9 |
| Oncostatin M | OSM | NM_020530.3 | 2.8 |
| Multiple EGF-like-domains 9 | MEGF9 | NM_001080497.1 | 2.8 |
| Dysferlin, limb girdle muscular dystrophy 2B (autosomal recessive) | DYSF | NM_003494.2 | 2.8 |
| Matrix metallopeptidase 25 | MMP25 | NM_022468.4 | 2.7 |
| Ring finger 144B | IBRDC2 | NM_182757.2 | 2.7 |
| Egf-like module containing, mucin-like, hormone receptor-like 3 | EMR3 | NM_032571.3 | 2.7 |
| Yip1 domain family, member 4 | YIPF4 | NM_032312.2 | 2.6 |
| Plexin domain containing 2 | PLXDC2 | NM_032812.7 | 2.6 |
| ST6 (alpha-N-acetyl-neuraminyl-2,3-beta-galactosyl-1,3)-N-acetylgalactosaminide alpha-2,6-sialyltransferase 2 | ST6GALNAC2 | NM_006456.1 | 2.6 |
| Coagulation factor V (proaccelerin, labile factor) | F5 | NM_000130.4 | 2.6 |
| Ectonucleoside triphosphate diphosphohydrolase 1 | ENTPD1 | NM_001098175.1 | 2.6 |
| Netrin G2 | NTNG2 | NM_032536.1 | 2.6 |
| CD55 molecule, decay accelerating factor for complement (Cromer blood group) | CD55 | NM_000574.2 | 2.6 |
| Carboxypeptidase D | CPD | NM_001304.3 | 2.6 |
| Transmembrane protein 184B | TMEM184B | NM_012264.3 | 2.5 |
| Cytidine deaminase | CDA | NM_001785.2 | 2.5 |
| Solute carrier family 22 (organic cation/ergothioneine transporter), member 4 | SLC22A4 | NM_003059.2 | 2.5 |
| TIMP metallopeptidase inhibitor 2 | TIMP2 | NM_003255.4 | 2.5 |
| Olfactory receptor, family 10, subfamily G, member 3 | OR10G3 | NM_001005465.1 | 2.5 |
| Endoplasmic reticulum-golgi intermediate compartment (ERGIC) 1 | ERGIC1 | NM_020462.1 | 2.5 |
| Diacylglycerol O-acyltransferase homolog 2 (mouse) | DGAT2 | NM_032564.2 | 2.5 |
| Solute carrier family 11 (proton-coupled divalent metal ion transporters), member 1 | SLC11A1 | NM_000578.3 | 2.5 |
| UDP-Gal:betaGlcNAc beta 1,4- galactosyltransferase, polypeptide 5 | B4GALT5 | NM_004776.2 | 2.5 |
| Plexin C1 | PLXNC1 | NM_005761.1 | 2.4 |
| Aquaporin 9 | AQP9 | NM_020980.2 | 2.4 |
| Transmembrane protein 55A | TMEM55A | NM_018710.1 | 2.4 |
| Similar to VAMP (vesicle-associated membrane protein)-associated protein A, 33kDa | ABHD2 | NM_152924.3 | 2.4 |
| Solute carrier family 22, member 17 | SLC22A17 | NM_016609.3 | 2.4 |
| STEAP family member 4 | STEAP4 | NM_024636.2 | 2.4 |
| Thioredoxin domain containing 13 | TXNDC13 | NM_021156.2 | 2.4 |
| Cytoskeleton-associated protein 4 | CKAP4 | NM_006825.2 | 2.4 |
| Alkaline phosphatase, liver/bone/kidney | ALPL | NM_000478.3 | 2.3 |
| Sialic acid binding Ig-like lectin 5 | SIGLEC5 | NM_003830.1 | 2.3 |
| Formyl peptide receptor 2 | FPRL1 | NM_001005738.1 | 2.3 |
| Bestrophin 1 | BEST1 | NM_004183.2 | 2.3 |
| Adenylate cyclase 4 | ADCY4 | NM_139247.2 | 2.3 |
| Mannosidase, alpha, class 2A, member 2 | MAN2A2 | NM_006122.2 | 2.3 |
| Arachidonate 5-lipoxygenase-activating protein | ALOX5AP | NM_001629.2 | 2.3 |
| C-type lectin domain family 7, member A | CLEC7A | NM_197948.2 | 2.3 |
| Signal-regulatory protein beta 1 | SIRPB1 | NM_006065.1 | 2.3 |
| Transmembrane protein 120A | TMEM120A | NM_031925.1 | 2.2 |
| Carcinoembryonic antigen-related cell adhesion molecule 3 | CEACAM3 | NM_001815.2 | 2.2 |
| Ecotropic viral integration site 2A | EVI2A | NM_014210.2 | 2.2 |
| Myelin protein zero-like 2 | EVA1 | NM_005797.2 | 2.2 |
| ATP-binding cassette, sub-family G (WHITE), member 1 | ABCG1 | NM_016818.2 | 2.2 |
| Cysteine-rich secretory protein LCCL domain containing 2 | CRISPLD2 | NM_031476.2 | 2.2 |
| Major histocompatibility complex, class I, C | HLA-C | NM_002117.4 | 2.2 |
| Chromosome 20 open reading frame 3 | C20orf3 | NM_020531.2 | 2.2 |
| Solute carrier family 16, member 3 (monocarboxylic acid transporter 4) | SLC16A3 | NM_004207.2 | 2.2 |
| NFAT activating protein with ITAM motif 1 | NFAM1 | NM_145912.5 | 2.2 |
| Platelet-activating factor receptor | PTAFR | NM_000952.3 | 2.2 |
| Syntaxin binding protein 5 (tomosyn) | STXBP5 | NM_139244.2 | 2.2 |
| Solute carrier family 12 (potassium/chloride transporters), member 6 | SLC12A6 | NM_001042496.1 | 2.2 |
| CKLF-like MARVEL transmembrane domain containing 2 | CMTM2 | NM_144673.2 | 2.2 |
| Interleukin 17 receptor A | IL17RA | NM_014339.4 | 2.2 |
| Chondroitin sulfate N-acetylgalactosaminyltransferase 2 | GALNACT-2 | NM_018590.3 | 2.1 |
| Formyl peptide receptor 1 | FPR1 | NM_002029.3 | 2.1 |
| Syntaxin 3 | STX3 | NM_004177.3 | 2.1 |
| Chemokine (C-X-C motif) ligand 16 | CXCL16 | NM_022059.1 | 2.1 |
| Pecanex homolog (Drosophila) | PCNX | NM_014982.2 | 2.1 |
| C-type lectin domain family 5, member A | CLEC5A | NM_013252.2 | 2.1 |
| Solute carrier family 22, member 15 | SLC22A15 | NM_018420.1 | 2.1 |
| Fc fragment of IgG, low affinity IIa, receptor (CD32) | FCGR2A | NM_021642.2 | 2.1 |
| Prokineticin 2 | PROK2 | NM_021935.2 | 2.1 |
| Leukocyte immunoglobulin-like receptor, subfamily B (with TM and ITIM domains), member 3 | LILRB3 | NM_006864.2 | 2.1 |
| Endoplasmic reticulum-golgi intermediate compartment (ERGIC) 1 | ERGIC1 | NM_001031711.1 | 2.1 |
| Colony stimulating factor 3 receptor (granulocyte) | CSF3R | NM_156038.2 | 2.1 |
| Coagulation factor II (thrombin) receptor-like 1 | F2RL1 | NM_005242.3 | 2.1 |
| Myelin protein zero-like 1 | MPZL1 | NM_003953.4 | 2.1 |
| Cholinergic receptor, nicotinic, alpha 10 | CHRNA10 | NM_020402.2 | 2.1 |
| Sortilin-related receptor, L(DLR class) A repeats-containing | SORL1 | NM_003105.3 | 2.1 |
| Syntaxin 7 | STX7 | NM_003569.1 | 2.1 |
| Anterior pharynx defective 1 homolog B (C. elegans) | APH1B | NM_031301.2 | 2.1 |
| ST3 beta-galactoside alpha-2,3-sialyltransferase 4 | ST3GAL4 | NM_006278.1 | 2.0 |
| Purinergic receptor P2X, ligand-gated ion channel, 1 | P2RX1 | NM_002558.2 | 2.0 |
| Interleukin 6 receptor | IL6R | NM_000565.2 | 2.0 |
| Solute carrier family 31 (copper transporters), member 2 | SLC31A2 | NM_001860.2 | 2.0 |
| Solute carrier family 2 (facilitated glucose transporter), member 3 | SLC2A3 | NM_006931.1 | 2.0 |
| ELOVL family member 5, elongation of long chain fatty acids (FEN1/Elo2, SUR4/Elo3-like, yeast) | ELOVL5 | NM_021814.3 | 2.0 |
| Fucosyltransferase 7 (alpha (1,3) fucosyltransferase) | FUT7 | NM_004479.2 | 2.0 |
| Serine incorporator 1 | SERINC1 | NM_020755.2 | 2.0 |
| Neuroplastin | NPTN | NM_012428.2 | 2.0 |
| Multiple C2 domains, transmembrane 2 | MCTP2 | NM_018349.2 | 2.0 |
| Cathelicidin antimicrobial peptide | CAMP | NM_004345.3 | 2.0 |
| Transmembrane 6 superfamily member 1 | TM6SF1 | NM_023003.2 | 2.0 |
| Interleukin 8 receptor, alpha | IL8RA | NM_000634.2 | 2.0 |
| Zinc finger, DHHC-type containing 18 | ZDHHC18 | NM_032283.1 | 2.0 |
| Signaling lymphocytic activation molecule family member 1 | SLAMF1 | NM_003037.1 | -2.0 |
| Lectin, galactoside-binding, soluble, 3 binding protein | LGALS3BP | NM_005567.2 | -2.0 |
| Single immunoglobulin and toll-interleukin 1 receptor (TIR) domain | SIGIRR | NM_021805.1 | -2.0 |
| CD247 molecule | CD247 | NM_000734.2 | -2.0 |
| ORM1-like 3 (S. cerevisiae) | ORMDL3 | NM_139280.1 | -2.0 |
| Solute carrier family 2 (facilitated glucose transporter), member 6 | SLC2A6 | NM_017585.2 | -2.0 |
| CD96 molecule | CD96 | NM_198196.2 | -2.0 |
| Leucine rich repeat containing 33 | LRRC33 | NM_198565.1 | -2.1 |
| Major histocompatibility complex, class II, DP beta 1 | HLA-DPB1 | NM_002121.4 | -2.1 |
| Solute carrier family 2, (facilitated glucose transporter) member 8 | SLC2A8 | NM_014580.3 | -2.1 |
| Family with sequence similarity 38, member A | FAM38A | NM_014745.1 | -2.1 |
| Aminolevulinate, delta-, synthase 2 | ALAS2 | NM_001037967.1 | -2.1 |
| G protein-coupled receptor 68 | GPR68 | NM_003485.3 | -2.1 |
| Zeta-chain (TCR) associated protein kinase 70kDa | ZAP70 | NM_001079.3 | -2.1 |
| Granulysin | GNLY | NM_006433.2 | -2.1 |
| Chemokine (C-X-C motif) receptor 3 | CXCR3 | NM_001504.1 | -2.1 |
| CD2 molecule | CD2 | NM_001767.2 | -2.1 |
| G protein-coupled receptor 18 | GPR18 | NM_001098200.1 | -2.1 |
| Perforin 1 (pore forming protein) | PRF1 | NM_005041.4 | -2.2 |
| Chemokine (C-C motif) ligand 5 | CCL5 | NM_002985.2 | -2.2 |
| Granulysin | GNLY | NM_012483.1 | -2.2 |
|  | CCL4L2 | NM_207007.2 | -2.2 |
| Fc receptor-like 3 | FCRL3 | NM_001024667.1 | -2.2 |
| Fc fragment of IgE, high affinity I, receptor for; alpha polypeptide | FCER1A | NM_002001.2 | -2.2 |
| Integrin, beta 7 | ITGB7 | NM_000889.1 | -2.3 |
| CD8a molecule | CD8A | NM_171827.2 | -2.3 |
| CD3g molecule, gamma (CD3-TCR complex) | CD3G | NM_000073.1 | -2.3 |
| CD6 molecule | CD6 | NM_006725.2 | -2.3 |
| CD81 molecule | CD81 | NM_004356.3 | -2.3 |
| G protein-coupled receptor 56 | GPR56 | NM_201524.1 | -2.3 |
| Transforming growth factor, beta receptor III | TGFBR3 | NM_003243.2 | -2.3 |
| Solute carrier family 4, anion exchanger, member 1 (erythrocyte membrane protein band 3, Diego blood group) | SLC4A1 | NM_000342.2 | -2.3 |
| Interleukin 32 | IL32 | NM_001012636.1 | -2.3 |
| Family with sequence similarity 62 (C2 domain containing), member A | FAM62A | NM_015292.1 | -2.4 |
| Carbohydrate (chondroitin 4) sulfotransferase 12 | CHST12 | NM_018641.3 | -2.4 |
| Chemokine (C-X3-C motif) receptor 1 | CX3CR1 | NM_001337.3 | -2.4 |
| Natural cytotoxicity triggering receptor 3 | NCR3 | NM_147130.1 | -2.5 |
| Interleukin 2 receptor, beta | IL2RB | NM_000878.2 | -2.5 |
| LFNG O-fucosylpeptide 3-beta-N-acetylglucosaminyltransferase | LFNG | NM_001040167.1 | -2.6 |
| Killer cell lectin-like receptor subfamily G, member 1 | KLRG1 | NM_005810.3 | -2.6 |
| Platelet factor 4 variant 1 | PF4V1 | NM_002620.2 | -2.8 |
| Lymphocyte-activation gene 3 | LAG3 | NM_002286.4 | -3.0 |
| Calcium channel, voltage-dependent, gamma subunit 6 | CACNG6 | NM_031897.2 | -3.1 |
| Natural killer cell group 7 sequence | NKG7 | NM_005601.3 | -3.1 |
| G protein-coupled receptor 114 | GPR114 | NM_153837.1 | -3.2 |
| CD244 molecule, natural killer cell receptor 2B4 | CD244 | NM_016382.2 | -3.2 |
